# B fibers are the best predictors of cardiac activity during Vagus nerve stimulation: Qing, vagal B fiber activation and cardiac effects

**DOI:** 10.1186/s42234-018-0005-8

**Published:** 2018-04-20

**Authors:** Kurt Y. Qing, Kelsey M. Wasilczuk, Matthew P. Ward, Evan H. Phillips, Pavlos P. Vlachos, Craig J. Goergen, Pedro P. Irazoqui

**Affiliations:** 1 Biomedical Engineering, Purdue University, West Lafayette, IN USA; 2 Indiana University School of Medicine, Indianapolis, IN USA; 3 Mechanical Engineering, Purdue University, West Lafayette, IN USA

**Keywords:** Vagus nerve stimulation, Heart failure, Closed-loop stimulation, [110], [85], [171], [106], [31]

## Abstract

**Background:**

Vagus nerve stimulation (VNS) is a promising therapy for many neurologic and psychiatric conditions. However, determining stimulus parameters for individual patients is a major challenge. The traditional method of titrating stimulus intensity based on patient perception produces highly variable responses. This study explores using the vagal response to measure stimulation dose and predict physiological effect. Clinicians are investigating the use of VNS for heart failure management, and this work aims to correlate cardiac measures with vagal fiber activity.

**Results:**

By recording vagal activity during VNS in rats and using regression analysis, we found that cardiac activity across all animals was best correlated to the activation of a specific vagal fiber group. With conduction velocities ranging from 5 to 10 m/s, these fibers are classified as B fibers (using the Erlanger-Gasser system) and correspond to vagal parasympathetic efferents.

**Conclusions:**

B fiber activation can serve as a standardized, objective measure of stimulus dose across all subjects. Tracking fiber activation provides a more systematic way to study the effects of VNS and in the future, may lead to a more consistent method of therapy delivery.

**Electronic supplementary material:**

The online version of this article (10.1186/s42234-018-0005-8) contains supplementary material, which is available to authorized users.

## Background

Vagus nerve stimulation (VNS) involves stimulating the cervical vagus nerve with electrical currents, activating nerve fibers. This therapy has been successful in treating patients suffering from refractory epilepsy (Ben-Menachem 2002) and depression (Sackeim et al. 2001), as well as has shown potential in treating other neurologic and psychiatric conditions (Groves and Brown 2005). The vagus nerve also exerts a strong influence on the heart (Cohn 1912; Dale 1934), which has important implications for treating cardiac conditions.

Heart failure presents as progressive structural remodeling of the heart tissue and deterioration of its function, processes that can be irreversible and difficult to control. Consequently, despite recent advances in therapy, mortality and morbidity remain high, with 5-year mortality still being approximately 50% (Mozaffarian et al. 2014). In search of alternative treatments, clinicians have been investigating the use of VNS for managing heart failure (Schwartz et al. 2008; De Ferrari et al. 2011; Zannad et al. 2015; Hauptman et al. 2012). Vagal innervation of the heart is asymmetrical in nature, such that the right vagus affects the heart rate more than the left (Cohn 1912; Randall and Armour 1974; Ardell and Randall 1986). Therefore, unlike in epilepsy and depression, where VNS targets the left vagus to avoid cardiac side effects, the right vagus is the target for heart failure.

Despite its initial promise, VNS produced inconclusive results in clinical studies (Schwartz et al. 2015). In two earlier trials, heart function and quality of life for participants receiving VNS improved significantly (Schwartz et al. 2008; De Ferrari et al. 2011). However, in the recent NECTAR-HF randomized clinical trial, while quality of life did improve, there was no significant improvement in heart function (Zannad et al. 2015). In addition, while patients overall responded to treatment in the two earlier studies, response varied despite efforts to ensure that each patient received adequate stimulation (Schwartz et al. 2008; De Ferrari et al. 2011).

Stimulation dosing remains a major challenge. “Adequate” stimulation is determined as between a level perceivable by the patient and a level intolerable due to side effects, both of which are subjective and inexact. In current standard practice (Labiner and Ahern 2007), VNS parameters are set individually and tuned periodically for each patient, a method also adopted by the heart failure clinical studies. Effective treatment theoretically relies on the activation of parasympathetic nerve fibers that innervate the heart, but the standard dosing method does not incorporate any measurement of fiber activation. As a result, there is no consistent way to determine how parameters are affecting the same patient over time or to compare the effect of stimulation across all patients. Thus, the difficulty of determining stimulus parameters and the lack of physiological response data create significant obstacles for treating heart failure (Schwartz et al. 2015).

Nerve activation ultimately governs the effects of VNS. Activation of the vagal parasympathetic efferent fibers to the heart can directly decrease heart rate and contractile force (Löffelholz and Pappano 1985; Higgins et al. 1973). Activation of afferent fibers can decrease sympathetic activity as well (Schwartz et al. 2015). The results are similar to the effects of standard therapy for heart failure, which focuses on decreasing the blood pressure, heart rate, and energy demands of the heart (Hunt et al. 2009). Moreover, VNS can potentially correct the defective parasympathetic activity observed in patients with heart failure (De Ferrari et al. 2011; Zannad et al. 2015; Eckberg et al. 1971; Thayer et al. 2010), an effect not addressed by standard medical therapy.

Because of factors such as electrode-tissue interface and nerve sensitivity, stimulation does not perfectly correlate with activation. Therefore, in this work, we reconsider the dosing-by-the-stimulus notion and hypothesize that the effects on the heart can be better correlated to nerve activation. We aim to show that vagal fiber activation, rather than stimulus parameters, provides a consistent measure of stimulation dose and a predictor of cardiac effects.

Our study focuses on the acute effect of VNS on heart activity, with the assumption that decreasing the heart rate is a marker of the potential benefits of therapy. The mechanism of VNS in treating heart failure and the potential chronic benefits of VNS on heart health are not considered herein.

## Methods

### Animals and surgery

All procedures were approved by the Institutional Animal Care and Use Committee (IACUC) and adhered to the NIH *Guide for the Care and Use or Laboratory Animals (Eighth Edition)*. The experimental animals were adult female Long-Evans rats (250-300 g). Isoflurane (induction: 5% in 2 L/min O_2_; maintenance: 0.5–1.5%) was delivered as anesthesia during the entire procedure, except one animal received ketamine/xylazine instead (75–95 mg/kg ketamine + 5 mg/kg xylazine intraperitoneal injections). Butorphanol (1.5 mg/kg per 4 h of surgery) was injected subcutaneously for analgesia.

A midline cervical incision was made, and the right carotid sheath between the infrahyoid and the sternocleidomastoid muscles was exposed. The omohyoid muscle was left intact. Due to the complexity of the rat cervical anatomy, it was difficult to expose a long segment of the vagus nerve without damaging nearby structures and potential vagal branches. Therefore, only short segments of vagus nerve above and below the omohyoid were exposed and then isolated from inside the carotid sheath.

After peeling away epineurium, two 2-lead cuff electrodes were wrapped around the nerve. The recording cuff was placed above the omohyoid muscle, near the carotid bifurcation, and the stimulation cuff was placed below the omohyoid. The typical distance between the two cuffs was 7.0 mm (range 6.0–8.0 mm). Similar procedures can be found in earlier publications (Ward et al. 2015; Qing et al. 2015). Because the nerve potentially branches in the neck, the distal cuff always served as the stimulation cuff. This configuration ensures that all nerve fibers in the stimulation cuff were wrapped by the proximal recording cuff and that the stimulation cuff is far away from the carotid sinus to avoid activating the baroreflex afferents.

### Electrodes

The cuffs were custom made. The electrode leads were braided platinum-iridium microwires (90% platinum, 10% iridium, Fort Wayne Metals), and the cuff material was silicone tubing (AM Systems 806700). For each cuff, the leads were spaced 1 mm apart and exposed only inside the cuff, with an estimated contact area of 0.011cm^2^ for each lead. These electrodes were characterized in detail in an earlier study (Qing et al. 2015).

### Experimental setup

The stimulus waveforms were generated digitally using Matlab and then converted into an analog signal by a data acquisition system (DAQ, National Instruments, USB-635×). A custom-made Howland current pump (±10 V power supply) then converted the voltage signal into a current waveform and passed to the distal cuff (details about the current pump can be found in a prior publication (Qing et al. 2015)). During stimulation, the vagal response at the proximal cuff was conditioned using a Grass P511 preamplifier (1 Hz – 10 kHz passband, 1000 – 2000× gain, and 60 Hz notch) and acquired by the same DAQ at 25 kHz.

**Fig. 1:**
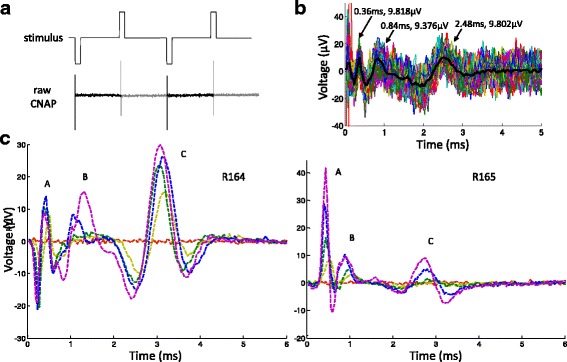
Vagal nerve response to stimulus. **a** Sample stimulus waveform (not to scale) and raw vagal recording. The darker segment of the recording represents the signal during the cathodic phase; the lighter segment represents the signal during the identical anodic phase half a period later. Two periods of alternating phases are shown. The spikes are due to stimulus artifacts, whose magnitudes were on the order of mV to V, depending on the stimulus intensity. **b** Sample processed CNAP. Summing the cathodic phase with the anodic eliminated the artifact. The summed segments (dotted traces) were then averaged to produce the estimated CNAP (thick, solid trace) for the trial. The three prominent peaks are labeled. **c** Sample CNAP series from the same stimulus waveform at different current amplitudes. As current increased, the peak heights (magnitude on the order of μV) increased as well. Peak designations are annotated. Data from two animals (R164 and R165) are presented to demonstrate variations in CNAP morphology

All stimuli were charge-balanced, cathode-leading, alternating monophasic waveforms (Ward et al. 2015), which involve a cathodic phase followed by an identical anodic phase at half the period. Because of the limited length of the cervical vagus nerve in rats (6-7 mm typical separation between stimulation and recording electrodes), the conduction distance between the stimulation and recording electrodes was too short to allow the stimulus artifact to fully separate from the vagal response. With alternating monophasic waveforms, the artifact was cancelled by summing the cathodic and anodic phases of the recorded signal (see Fig. 1). In our experiments, pulse width ranged from 40 to 200 μs, and current amplitude was below 1.5 mA. With these parameters, only the cathode phase elicited a vagal response (please see our prior publication (Ward et al. 2015) for more details).

Electrocardiograms (ECG) were recorded using surface electrodes with snap-on connectors (Covidien BRD H124SG) in bipolar lead II configuration. Conductive gel was applied to shaven skin to improve contact. ECG was also conditioned with a Grass preamplifier and acquired with the same DAQ, using the same settings as for the nerve. Similar experimental setups were used in earlier studies (Ward et al. 2015; Qing et al. 2015).

For ultrasound imaging, short-axis images of the left ventricle (LV) were acquired before, during, and after stimulation using a high-resolution system (Vevo2100, FUJIFILM VisualSonics) with a linear array, 256-element transducer optimized for rat imaging (MS250; 13-24 MHz frequency range with 21 MHz center frequency). Care was taken to avoid shadows from the sternum and ribs and to minimize interference from papillary muscles, chordae tendineae, and valves. We acquired brightness-mode (100 frames/s; 9-s acquisitions) and motion-mode (15-s acquisitions) images to track wall motion.

### Data processing

Vagal and ECG recordings were analyzed using Matlab. In the vagal signal, after cancelling stimulus artifacts and averaging the response, the compound nerve action potential (CNAP) became apparent (see Fig. 1b). Though CNAP morphology differed among animals, at least three major distinct peaks were always observed. The peaks were classified as A, B, and C fiber responses based on conduction velocity (calculated by dividing the conduction distance by peak latency), according to the Erlanger-Gasser classification system (Gasser 1941).

The morphology of the CNAP peaks varied among animals and even within the same animal. The peak complexes tended to be biphasic, even triphasic, and there was some overlap of the peak complexes (see Fig. 1c). Some peak complexes had larger negative components. We considered using area under the peak and peak-to-peak magnitude to represent the fiber response magnitude, but due to overlapping peak complexes, it was difficult to consistently assign the peak components. Because the peaks were always very distinct, the peak magnitude was chosen to estimate the fiber response.

In the raw ECG, stimulus artifacts were the dominating feature (see Additional file 1: Figure S1). The artifacts were removed by a 100- to 200-point moving average filter. Generally at higher stimulus intensities, the artifacts were larger and more problematic, requiring more smoothing. R peaks were manually marked for each trial, and then instantaneous heart rate was calculated using intervals between R peaks. The instantaneous values during the final 5 s of the 10-s stimulus were averaged to produce the heart rate during stimulation. The animals under anesthesia were breathing roughly once every 2 s. Averaging 5 s of heart rate data accounted for breathing-related variations, as well as potential ectopic beats. See Additional file 1: Figure S1 for ECG processing.

Ultrasound data were analyzed with Vevo LAB software (v1.6; FUJIFILM VisualSonics). Using the short-axis LV Trace Tool, we traced the anterior and posterior endocardial walls of the LV over two to three cardiac cycles during inspiration and expiration. Systolic and diastolic left ventricle volumes (LV Vs and LV Vd) were estimated from inner diameter measurements at peak systole (LVIDs) and end diastole (LVIDd) using the following equation:

$$ LV\ V=\left(7.0/\left(2.4+ LVID\right)\right)\ast {LVID}^3 $$

Then stroke volume (SV), ejection fraction (EF), and fractional shortening (FS) were calculated using the following equations:

$$ \mathrm{SV}=\mathrm{LV}\ \mathrm{Vd}\hbox{--} \mathrm{LV}\ \mathrm{Vs} $$

$$ \mathrm{EF}=\left(\mathrm{LV}\ \mathrm{Vd}\hbox{--} \mathrm{LV}\ \mathrm{Vs}\right)/\mathrm{LV}\ \mathrm{Vd} $$

$$ \mathrm{FS}=\left(\mathrm{LVIDd}\hbox{--} \mathrm{LVIDs}\right)/\mathrm{LVIDd} $$

Cardiac output (CO) was obtained by multiplying SV by heart rate. The values during inspiration and expiration were averaged to produce final estimations during stimulation. Anatomical M-mode images (converted from B-mode data) and standard M-mode images were used for analysis.

Nerve, ECG, and ultrasound data were analyzed separately by three individuals. Since all stimulation trials were randomized by software, the analysis team was blinded to stimulus parameters and trial order. In cases of large discrepancies, the data were analyzed together.

### Stimulation waveforms and parameters

All of the waveforms were rectangular in shape, but different pulse widths were used. The shortest pulse width was 40 μs, and the longest was 200 μs. In addition, some waveforms utilized bursts of short pulses in place of standard rectangular pulses. These burst waveforms have been shown to produce different nerve activation profiles (Qing et al. 2015; Shepherd and Javel 1999). For our figures and analyses, data from all tested waveforms were pooled.

Stimulus frequency (pulse repetition) was set to 10 Hz initially, based on literature and our prior experiences. Later, the frequency was increased to 20 Hz. Both 10 and 20 Hz are within the range used in clinical settings, in established practice (Labiner and Ahern 2007) and investigative settings (Zannad et al. 2015).

Within the same time window, vagal fibers would fire more with 20 Hz stimulation than with 10 Hz, which leads to more acetylcholine release. As a result, the heart rate would change more quickly and reach a steady state faster (Carlson et al. 2012; Warner and Cox 1962; Warner and Russell 1969). At the same intensity, a higher frequency would also cause a greater reduction in heart rate, but the effect seems to be prominent only for frequencies below 10 Hz (Carlson et al. 2012). In our experiments, we observed the faster kinetics at 20 Hz and decided to use 20 Hz in later subjects to speed up data collection.

### Experimental design

The experiments followed a repeated measures design with trial randomization. Because the heart rate responds quickly to stimulation and recovers quickly as well, each stimulation trial consisted of a 10-s stimulus followed by at least 10 s of off-period for recovery. The stimulus parameters, including waveform and current amplitude, were randomized to control for potential order effects in nerve response and heart activity. The stimuli were then delivered by the software in an automated manner.

Before running the sequence of stimuli, the current amplitude values were determined empirically. The highest current for each waveform was set such that the heart rate dropped to 200 beats per minute (bpm), because atrioventricular conduction block tended to occur when heart rate drops below 200 bpm (see Additional file 1: Figure S2 for conduction block). The lowest current is 0, which produces no fiber activation. About 10 values in between were used (0%, 20% of max, etc.) so as to capture the full heart rate reduction profile for each waveform. Waveforms were selected based on the results of our prior VNS study (Qing et al. 2015).

There were no predetermined sample sizes. Data from all animals were included and presented.

### Statistical analysis

Statistical analysis was performed in SAS 9.4. The REG procedure was used to fit the multiple linear regression models. All data points were included without outlier omission.

## Results

### The effect of stimulation on the nerve

As the stimulus intensity increased, the CNAP peaks generally increased in height as well (see Fig. 1c), signifying higher fiber recruitment and more fibers firing action potentials. Fiber recruitment profiles were sigmoidal in shape, eventually reaching a plateau. Vagal fiber recruitment profiles and the effect of stimulus waveforms have been studied before (Qing et al. 2015; Tosato et al. 2006; Mollet et al. 2013; Castoro et al. 2011). A detailed analysis of the effect of individual stimulus parameters on fiber recruitment can be found in our prior work (Ward et al. 2015).

### The effect of stimulation on the heart

**Fig. 2:**
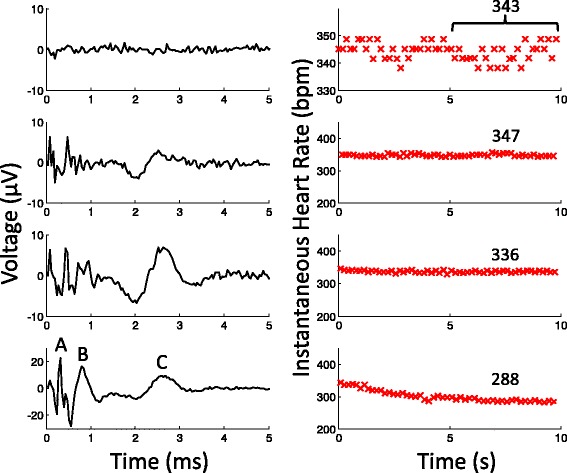
Sample CNAP with corresponding heart rate measurement. Left) Averaged CNAP from the same waveform at increasing intensities (charge per phase from top to bottom: 0, 6nC, 12nC, 24nC). Right) The instantaneous heart rate data during the 10-s trial. The heart rate values during the last 5 s were averaged to produce a representative value for the trial (annotated, in bpm). Note the different Y-axis scales in certain plots

The resting heart rate varied among the anesthetized rats (approximate range 300–380 bpm). The resting heart rate typically varied due to breathing, about a 10-bpm swing (example in Fig. 2), and sometimes drifted slowly over time as well.

For each animal, as the stimulus intensity increased, the heart rate decreased more and more from resting values (see Fig. 2). The amount of heart rate reduction and the rate of change were dependent on the stimulus and varied among animals. At very strong intensities, usually a charge per phase (Q) of > 40 nanocoulombs (nC), the heart rhythm even became irregular (see Additional file 1: Figure S2). After stimulation, the heart rate also recovered quickly—within a few seconds—to resting levels. More details on the kinetics of heart rate change to VNS can be found elsewhere (Schwartz et al. 2008; Warner and Russell 1969; Carlson et al. 1992).

**Fig. 3:**
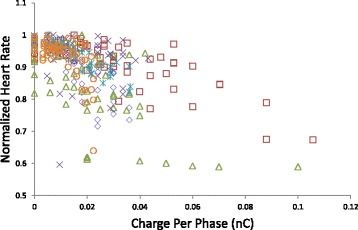
Stimulus intensity-heart rate profiles. The recorded normalized heart rate during stimulation trials was plotted against the stimulus charge per phase for all animals (*n* = 6, animals represented by different markers). There was generally an intensity threshold for eliciting a heart rate drop that differed among animals. The change in heart rate per change in charge also differed. Data from all animals were included without omissions, and data from all waveforms are pooled. Because stimulus charge per phase is the product of pulse width and amplitude, charge was used to represent stimulus intensity

When determining the proper stimulus intensity, the typical approach as seen in both animal experiments (Li et al. 2004; Zhang et al. 2009) and clinical studies (Schwartz et al. 2008; De Ferrari et al. 2011; Zannad et al. 2015) would be to fix the pulse width and then adjust the pulse amplitude for each subject. This way, the stimulus can be set to reach a target heart rate for each subject. However, different studies and groups tend to use different sets of parameters, and different devices have different parameter sets available. Overall, there is no consistent method to characterize the stimulus-heart rate relationship. To demonstrate using this typical approach, the normalized heart rate during our stimulation trials was plotted against the stimulus charge per phase in Fig. 3, which shows the combined data from all animals (*n* = 6). For normalization, each heart rate value was divided by the heart rate at rest.

While there seemed to be an overall decrease in heart rate with increasing charge, the relationship varied quite drastically from animal to animal, with some not as responsive to VNS as others. There are many potential outliers and influential points.

### The effect of nerve activation on heart rate

**Fig. 4:**
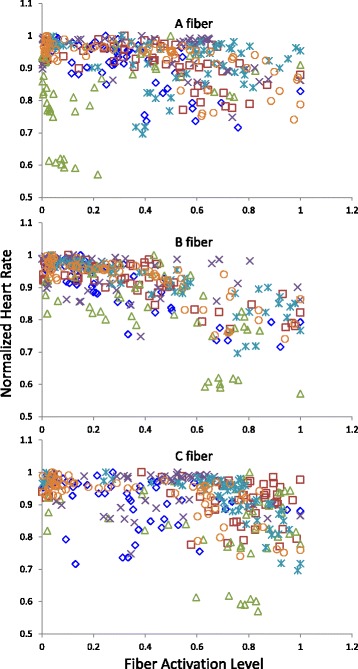
Vagal activation-heart rate profiles. The recorded heart rate was plotted against the resulting nerve fiber activation levels from the same stimulation trial. The same data set as in Fig. 3 was used. Data for all animals are presented (*n* = 6, animals represented by different markers). Both the heart rate and fiber activation data were normalized

Sample CNAP signals and their corresponding heart rate changes are shown in Fig. 2. To visualize the relationship between vagal activation and heart rate, the normalized heart rate during stimulation is plotted against the magnitudes of the different fiber peaks in Fig. 4. The plots for individual animals can be found in Additional file 1: Figure S3. To normalize the fiber response within each animal, each fiber response magnitude was divided by the maximal magnitude achieved for that fiber. Maximal fiber magnitude always occurred at higher stimulus intensities, and so a normalized value represents a fiber activation level relative to maximal activation.

By initial visual inspection, the reduction in heart rate seems to be linked to the B fiber magnitude. In some subjects, the heart rate appears to be correlated to A and C fiber activity as well, but the heart rate does not always change with early A and C fibers recruitment. In contrast, the heart rate decreases throughout the full range of B fiber activation, suggesting a graded response between B fiber activation and heart rate.

The stimulus-heart rate and activation-heart rate relationships were analyzed using the following multiple linear regression model:

$$ {HR}_{norm}=\beta \ast X+{\delta}_{animal}\ast {D}_{animal}+\varepsilon $$

*HR*_*norm*_ is the normalized heart rate. *β* represents the regression coefficient for the predictor *X*, which are the charge per phase (Q) and the normalized activation levels of right vagal A, B, and C fibers. *D*_*animal*_ is the dummy variable matrix for the animals, and *δ*_*animal*_ are the corresponding coefficients. *ε* is the error term.

Goodness of fit values were then compared for the different models to determine the best predictor of heart rate. The separate models cannot be integrated into a single model, because the variables *Q*, *A*, *B*, and *C* are not independent of each other.

Regression analysis indicated that decreasing HR_norm_ values are correlated with increasing Q, A, B, and C values (*p*-value < 0.0001 for all *β* values, see Table 1 and Additional file 2). With the highest coefficients of determination (R^2^) among the different models, B fiber activation produced the best fit (see Table 1). Furthermore, residuals for the models using Q, A, and C did not follow normal distributions, which suggest that the models with Q, A, and C are not appropriate predictors of heart rate (see Additional file 2).

**Table 1:** Regression coefficients and goodness of fit values for potential predictors of heart rate

Model	β	*p*-value	R^2^
HR_norm_-Q	−0.00297	< 0.0001	0.4745
HR_norm_-A	−0.08328	< 0.0001	0.2504
HR_norm_-B	−0.19671	< 0.0001	0.5851
HR_norm_-C	−0.08925	< 0.0001	0.2701

### The effects of nerve activation on heart activity

To further characterize how VNS affects the heart, VNS experiments were repeated with the addition of ultrasound imaging (*n* = 3 additional animals). A sample short-axis ultrasound recording of the ventricles during and after stimulation is provided (please see Additional file 3: stimulus started shortly before the video, note changes in the ECG trace). The playback speed was reduced to allow easier visualization of changes. A sample motion mode image depicting the estimation of ventricle wall dimensions from the ultrasound data is also provided (Additional file 1: Figure S4).

**Fig. 5:**
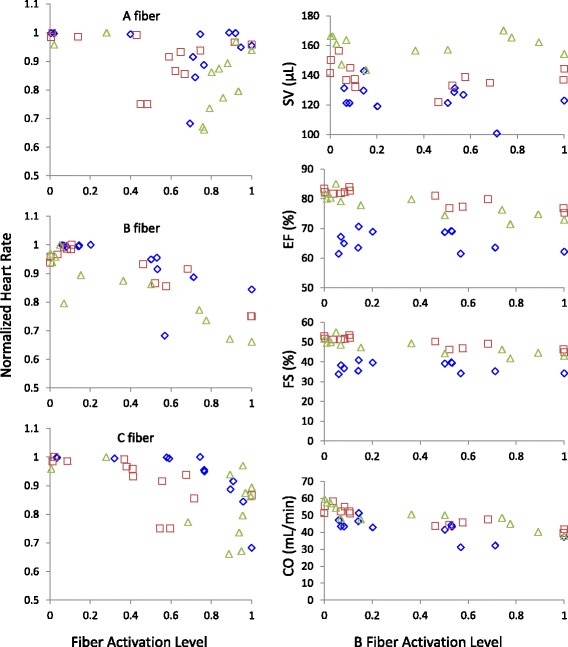
Vagal activation-heart activity profiles. Left) Normalized heart rate was plotted against normalized fiber activation level from the same stimulation trial. Right) Various measures of heart activity were calculated from ultrasound recordings and plotted against B fiber activation. The data in this figure are from a separate group of animals (*n* = 3, animals represented by different markers), presented without omission

Figure 5 summarizes the relationships between vagal activation and the resulting SV, EF, FS, and CO estimates, as well as normalized heart rate obtained from ECG. Consistent with the data from the first set of experiments, the normalized heart rate correlated best with normalized B fiber activation (results summarized in Table 2, also see Additional file 4). The same regression analysis was used.

**Table 2:** Regression coefficients and goodness of fit values for potential predictors of various measures of heart activity

Model	β	*p*-value	R^2^
HR_norm_-Q	−0.00410	< 0.0001	0.5511
HR_norm_-A	−0.07678	0.1603	0.1900
HR_norm_-B	−0.23462	< 0.0001	0.7459
HR_norm_-C	−0.17545	0.0005	0.4060
SV-B	−4.30763	0.3263	0.7371
EF-B	−6.50842	< 0.0001	0.8842
FS-B	−6.33329	< 0.0001	0.8875
CO-B	−14.25802	< 0.0001	0.7630

The relationship between the B fiber activation levels and the aforementioned measures of cardiac activity (SV, EF, FS, and CO) were then analyzed (results summarized in Table 2). The results suggested that EF, FS, and CO significantly decreased with increasing B fiber activation, while SV did not change significantly.

### The effect of stimulus waveform on the vagal-heart rate profile

**Fig. 6:**
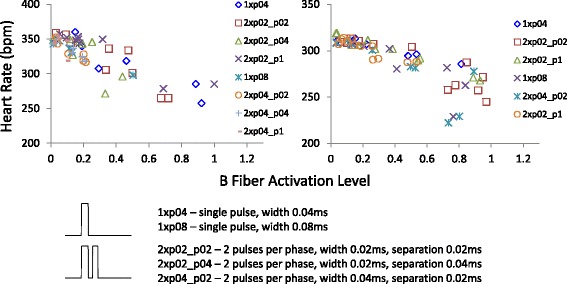
B fiber-heart rate profiles with different stimulus waveforms. Raw heart rate was plotted against B fiber activation with different stimulus waveforms denoted. Though different waveforms produced variable nerve activation patterns and variable effects on the heart rate, the B fiber and heart rate relationship appeared consistent. The two plots were from two different animals. Waveform legend is explained with example waveforms

Example vagal-heart rate profiles are shown in Fig. 6. Though the different waveforms produced different activation profiles (detailed analysis can be found in a prior publication (Qing et al. 2015)), the data points all followed the same trend. That the effect of VNS on heart rate is not dependent on the stimulus waveform further supports that the heart rate depends on B fiber activation and not the stimulus.

## Discussion

### Vagal activation versus vagal stimulation

Based on the existing knowledge of the vagus nerve and heart physiology, a direct correlation between vagal activation and heart activity should exist. Our results show that such a correlation indeed exists and is mediated by B fiber activation (Tables 1 and 2). Unlike arbitrary stimulus parameters, vagal activation is a standardized measure that applies to all subjects and provides a more reliable method to compare physiological response across subjects.

With stimulus-based dosing, the clinical studies reported that target heart rate reduction was achieved in less than half of the patients (Schwartz et al. 2008; De Ferrari et al. 2011). Often, before heart rate reduction was observed, the stimulator current reached maximal output, or the side effects became intolerable (Schwartz et al. 2008; De Ferrari et al. 2011). A retrospective study on VNS parameters and seizure reduction showed that patients who did not respond initially continued to receive increasing stimulation intensity (Labar 2004). With activation-based dosing, variables such as electrode interface and nerve sensitivity would be controlled.

In addition, it is known that acetylcholine released by vagal parasympathetic fibers is the agent causing a reduction in heart activity (Löffelholz and Pappano 1985; Higgins et al. 1973). Though reported values vary, the conduction velocity of vagal efferent parasympathetic fibers to the heart ranges roughly from 3 to 15 m/s (Jordan et al. 1982; McAllen and Spyer 1978). Based on the Erlanger-Gasser classification (Gasser 1941), these parasympathetic fibers fall into the B fiber category.

In our experiments, the vagal fibers that correlated best with the reduction in heart activity had conduction velocities in the approximate range of 5-10 m/s. These fibers that we designated as B fibers are exactly the preganglionic parasympathetic fibers that one would expect to be responsible for the cardiac effects.

Furthermore, an important implication of the independence of heart rate on waveform is that waveforms can be selected based on different efficiency and selectivity features. In theory, as long as the target level of B fiber activation is reached, the physiologic effect can be achieved. The waveform can potentially be optimized to produce the best efficiency and B fiber selectivity, so as to save energy and reduce side effects.

### Stimulation duty cycle

The effect of altering the duty cycle was not explored in detail in our experiments. Our effective duty cycle was 10s ON/10s OFF, or 50%. The length of the ON and OFF periods were chosen empirically such that, during the ON period, if vagal activation was sufficient, the heart rate would drop to a steady state value, and then during the OFF period, the heart rate would recover to resting value.

For treating epilepsy and depression, this ON/OFF type of stimulation is the norm (Labiner and Ahern 2007). For heart control, the ON/OFF type is not ideal because of heart rate ratcheting (Schwartz et al. 2008), where heart rate would drop when stimulation is ON and then rise when stimulation is OFF.

To avoid ratcheting, the two earlier clinical trials employed a strategy of timing each stimulus to the R peak, creating a clever duty-cycle-controlled paradigm with heart rate feedback (Schwartz et al. 2008; De Ferrari et al. 2011). This approach is elegant, but the actual stimulus and the effects are difficult to characterize and compare among patients. Interestingly, the recent randomized clinical trial switched to the more standard approach with a fixed duty cycle of 10s ON/50s OFF and frequency of 20 Hz. Again, stimulus parameters were set arbitrarily, making comparisons among different studies difficult.

### Implementing activation-based dosing

It would be possible to achieve better control of heart activity by stimulating at 100% duty cycle. Delivering a constant stimulus would result in a more constant level of nerve activation, and thus a more constant level of acetylcholine release and cardiac effect. There would be less ratcheting, and the intensity of each stimulus may be smaller and thus better tolerated. Furthermore, each level of effect can be attributed to a level of nerve fiber activation. Having an objective measure of physiological response that accounts for different stimulus waveforms, electrodes, and patient variability could decrease variability in therapeutic response and potentially increase response rate.

Vagal recording during stimulation is currently not standard procedure in clinical settings. The commercial implant lacks the capability to record from the nerve while it stimulates, and during clinical visits, stimulus parameters are adjusted without vagal recordings. Ideally, the hardware would eventually improve to allow direct nerve recording. In the meantime, even without new devices, surface electroneurograms can be recorded during stimulation when patients visit for parameter adjustment. Surface vagal recording during stimulation (Hammond et al. 1992) is not different from typical nerve conduction studies. ECG and ultrasound imaging, also common procedures, could be used during stimulation to characterize cardiac effects.

Furthermore, the mechanism for VNS in treating heart failure may not be simply driving parasympathetic output to the heart. Reducing sympathetic tone and improving parasympathetic tone may also be important (Hauptman et al. 2012; Hunt et al. 2009; Olshansky et al. 2008). Activation of the vagal baroreceptor afferent fibers can reduce sympathetic activity and increase parasympathetic activity, which would indirectly reduce heart rate, blood pressure, and force of contraction (Löffelholz and Pappano 1985; Higgins et al. 1973). Being afferent fibers, the baroreceptor fibers have a different conduction velocity and will likely present as a different peak in the CNAP. By tracking vagal activation in patients, the mechanism of VNS in treating heart failure can be studied in more detail.

## Conclusion

Our work showed that it is feasible to characterize the vagal response to various stimuli and correlate the vagal activation profile to effects on the heart. Further work is needed to determine the effect of different activation patterns on other physiologic parameters and ultimately to compare the long-term effects and endpoints. For heart failure specifically, it would be necessary to study whether targeting B fiber activation with the goal of overcoming adrenergic effects has a long-term impact on heart function and on survival. And it would be interesting to study whether vagal activation produces significant sympatholytic effects that may impact heart function and survival. Answering these questions can yield more insight into the mechanism of VNS for heart failure and improve the currently available treatments.

## Additional files

Additional file 1: **Figure S1.** ECG data processing and analysis. **Figure S2.** Example stimulus trial that resulted in conduction block. **Figure S3.** Right vagal fiber peak magnitude and heart rate data from all animals. **Figure S4.** LV trace of endocardial border over three cardiac cycles using VevoLab software (FUJIFILM VisualSonics). (DOCX 758 kb)

Additional file 2: Results from regression analysis of heart rate using SAS. (PDF 297 kb)

Additional file 3: Sample ultrasound recording during vagal stimulation. (MP4 12185 kb)

Additional file 4: Results from regression analysis of heart rate and sonographic data using SAS. (PDF 412 kb)

**Additional file 5:** ᅟ.
