# Supplementary material for: B fibers are the best predictors of cardiac activity during Vagus nerve stimulation: Qing, vagal B fiber activation and cardiac effects
Source: Bioelectron Med. 2018 Apr 20;4:5. doi: 10.1186/s42234-018-0005-8 (PMC7098216; doi:10.1186/s42234-018-0005-8)
Supplement: Supplementary file 1 — Figure S1. ECG data processing and analysis. Figure S2. Example stimulus trial that resulted in conduction block. Figure S3. Right vagal fiber peak magnitude and heart rate data from all animals. Figure S4. LV trace of endocardial border over three cardiac cycles using VevoLab software (FUJIFILM VisualSonics). (DOCX 758 kb) [file 42234_2018_5_MOESM1_ESM.docx]

**Figure S1. ECG data processing and analysis.** The 10-second raw ECG signal contains large spikes due to stimulus artifacts, which can be easily removed by smoothing. Though the ECG waveform is deformed slightly, smoothing does not affect detection of R peaks. R peaks are the most prominent feature (denoted by red x’s above each peak in the normalized ECG), and P and T waves are generally seen as well. All ECG trace and its R peaks are visualized and checked for accuracy. The instantaneous heart rate is estimated from the R-R intervals. In this example, the stimulus caused a drop in the heart rate.


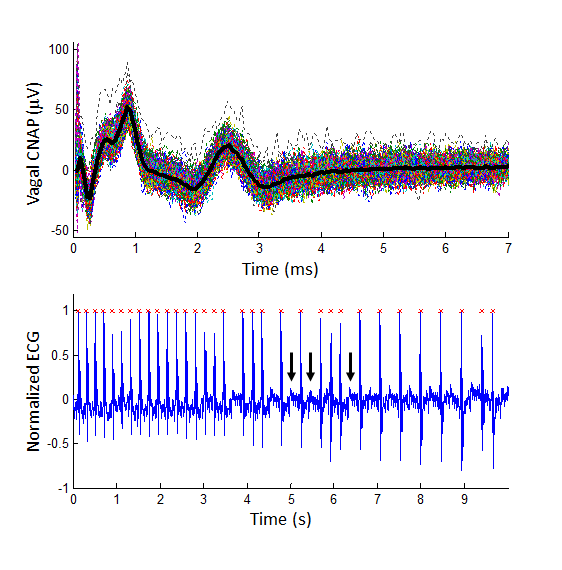


**Figure S2. Example stimulus trial that resulted in conduction block.** In the initial portion of the ECG segment, the heart rate is regular. As stimulation continues, conduction can be seen. The black arrows point to several P waves without a QRS complex. P-P intervals seem regular. The heart rate estimated from R peaks is very low and not representative of sinus rhythm, and these trials are omitted. Typically, the lower limit of heart rate during stimulation is around 200bpm; lower heart rates are usually due to conduction block.


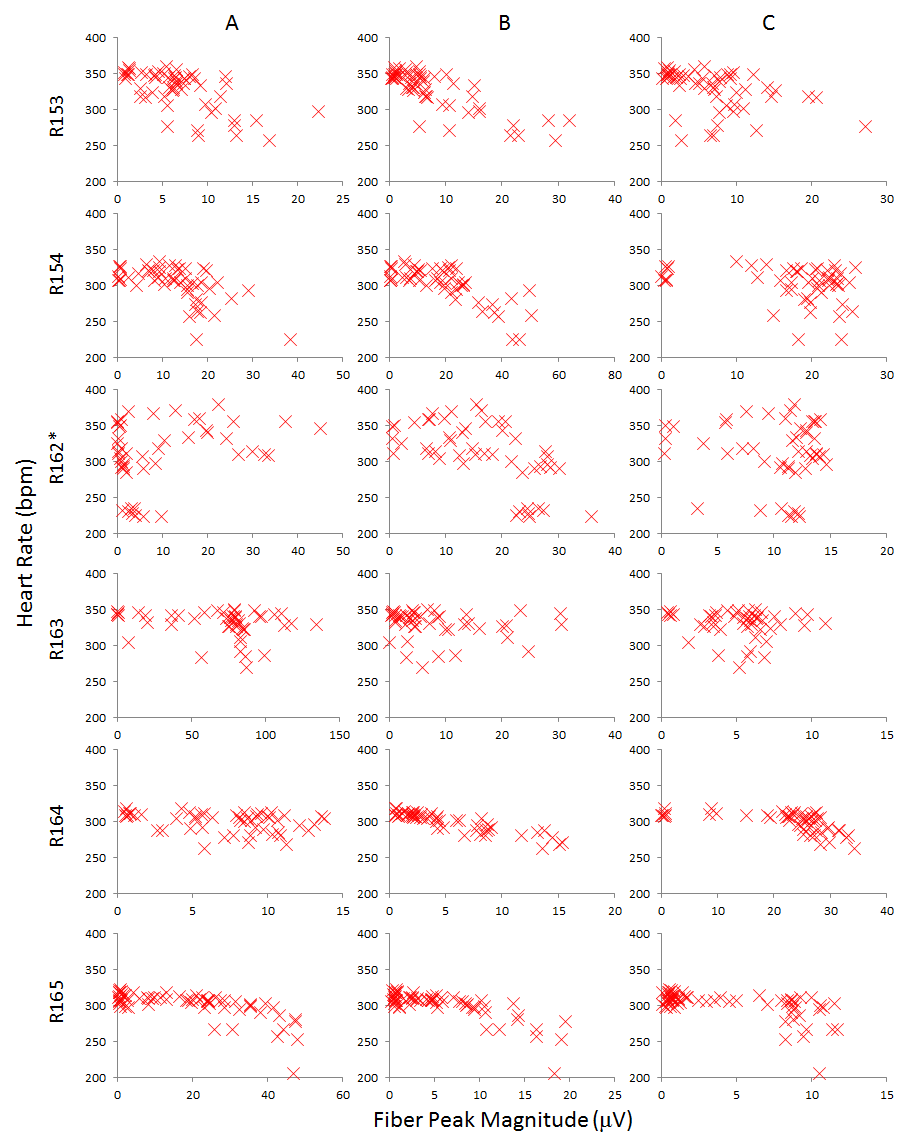


**Figure S3. Right vagal fiber peak magnitude and heart rate data from all animals.** Each row is data from a single animal, including all stimulus waveforms. The heart rate values during stimulation are plotted against A, B, and C fiber peak magnitudes in the different columns. *Note, heart rate from R162 is more variable because ketamine/xylazine injections were used for anesthesia instead of isoflurane.


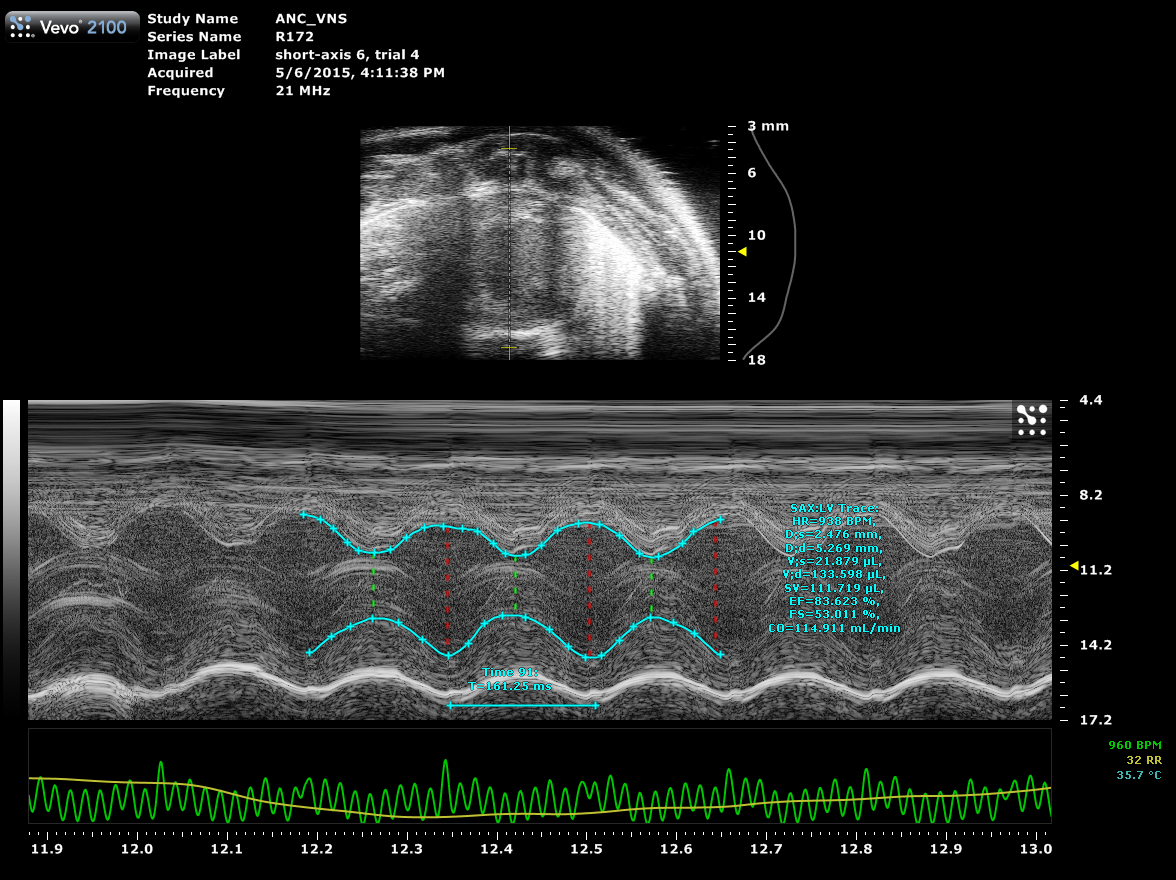


**Figure S4. LV trace of endocardial border over three cardiac cycles using VevoLab software (FUJIFILM VisualSonics).** The smaller image on top shows the anatomical view; the bottom image shows the M-mode data. The green trace represents the ECG, with heavy unprocessed stimulus artifact. The cyan trace represents manual tracing of LV inner wall for each trial. The cardiac activity estimates were automatically calculated based on the trace.
