# Supplementary material for: B fibers are the best predictors of cardiac activity during Vagus nerve stimulation: Qing, vagal B fiber activation and cardiac effects
Source: Bioelectron Med. 2018 Apr 20;4:5. doi: 10.1186/s42234-018-0005-8 (PMC7098216; doi:10.1186/s42234-018-0005-8)
Supplement: Supplementary file 2 — Results from regression analysis of heart rate using SAS. (PDF 297 kb) [file 42234_2018_5_MOESM2_ESM.pdf]

The SAS System

Obs	Rat	Q	A	B	C	HR_norm	SV	EF	FS	CO	R166dummy	R172dummy
1	R166	0.0	0.00535	0.06039	0.03239	0.99852	131.260	61.4470	33.7710	47.1893	1	0
2	R166	5.0	0.02041	0.06979	0.03556	0.99732	121.354	67.1808	38.2485	43.5753	1	0
3	R166	10.0	0.39867	0.08257	0.31984	0.99465	121.250	64.9660	36.6998	43.4215	1	0
4	R166	15.0	0.88792	0.20246	0.74473	1.00000	119.036	68.8875	39.5930	42.8580	1	0
5	R166	20.0	0.74587	0.14231	0.59209	0.99483	129.646	63.4928	35.5073	46.4363	1	0
6	R166	25.0	0.92030	0.14423	0.57908	0.99854	142.822	70.6230	40.8575	51.3468	1	0
7	R166	30.0	0.69519	0.56922	1.00000	0.68287	126.803	61.5230	34.1528	31.1757	1	0
8	R166	35.0	0.70960	0.53274	0.90895	0.91528	131.257	68.9973	39.4458	43.2543	1	0
9	R166	40.0	0.94707	0.50275	0.76651	0.94888	121.380	68.7243	39.1190	41.4678	1	0
10	R166	45.0	1.00000	0.53042	0.76438	0.95472	128.662	69.1768	39.7125	44.2259	1	0
11	R166	50.0	0.76370	0.71267	0.89573	0.88682	100.794	63.5750	35.2300	32.1825	1	0
12	R166	60.0	0.72046	1.00000	0.95845	0.84390	122.929	62.1208	34.2440	37.3505	1	0
13	R172	0.0	0.01173	0.10752	0.02200	1.00000	132.169	82.7970	52.2170	51.0218	0	1
14	R172	5.0	0.00531	0.10524	0.01206	0.98423	137.413	83.8790	53.5790	52.2097	0	1
15	R172	10.0	0.13906	0.08534	0.08581	0.98571	145.015	82.1685	51.7200	55.1805	0	1
16	R172	15.0	0.43028	0.06929	0.36870	0.99131	136.755	81.8165	51.2520	52.3333	0	1
17	R172	20.0	0.91618	0.03695	0.37952	0.96630	156.420	81.6950	51.3280	58.3485	0	1
18	R172	25.0	1.00000	0.00244	0.41000	0.95835	150.352	82.2075	51.8045	55.6239	0	1
19	R172	30.0	0.74558	0.00000	0.67493	0.93810	141.507	83.3640	53.0710	51.2451	0	1
20	R172	35.0	0.58997	0.68243	0.55711	0.91558	134.847	79.8325	49.1470	47.6610	0	1
21	R172	40.0	0.66879	0.57698	0.71459	0.85515	138.704	77.3395	46.7790	45.7885	0	1
22	R172	45.0	0.64872	0.46241	0.41247	0.93196	121.903	81.0085	50.2685	43.8569	0	1
23	R172	50.0	0.62204	0.52189	1.00000	0.86611	132.897	76.8545	46.2345	44.4338	0	1
24	R172	55.0	0.44858	1.00000	0.54508	0.75010	144.396	75.2225	44.8945	41.8119	0	1
25	R172	60.0	0.48311	0.99698	0.59676	0.75062	136.896	76.8670	46.3570	39.6677	0	1
26	R173	5.0	0.28085	0.04933	0.27878	1.00000	147.198	85.0440	55.0285	54.3655	0	0
27	R173	25.0	0.85863	0.74140	0.68526	0.77253	170.179	76.2935	46.1825	48.5559	0	0
28	R173	15.0	1.00000	0.01148	0.89345	0.93848	166.187	79.9440	49.5775	57.6028	0	0
29	R173	32.5	0.76535	1.00000	0.88794	0.66178	154.410	72.9800	42.9735	37.7406	0	0
30	R173	17.5	0.93502	0.06949	0.95593	0.79528	163.735	79.0720	48.6940	48.0935	0	0
31	R173	10.0	0.92272	0.00229	0.95693	0.96977	166.359	81.7980	51.5445	59.5849	0	0
32	R173	0.0	0.02082	0.02716	0.00666	0.95800	161.142	80.3135	49.9415	57.0158	0	0
33	R173	30.0	0.75791	0.89194	0.94972	0.67151	162.247	74.7455	44.5895	40.2394	0	0
34	R173	20.0	0.88070	0.15301	1.00000	0.89390	143.593	77.8110	47.2730	47.4075	0	0
35	R173	22.5	0.83798	0.36393	0.96928	0.87437	156.708	79.8320	49.4045	50.6067	0	0
36	R173	27.5	0.79134	0.77546	0.93804	0.73637	165.471	71.4520	41.7965	45.0031	0	0
37	R173	25.0	0.80145	0.50278	0.99661	0.86200	157.215	74.3525	44.2165	50.0526	0	0

The SAS System

The REG Procedure
Model: MODEL1
Dependent Variable: HR_norm

**Number of Observations Read**	37
**Number of Observations Used**	37

Analysis of Variance					
**Source**	**DF**	**Sum of Squares**	**Mean Square**	**F Value**	**Pr > F**
**Model**	3	0.21432	0.07144	13.51	<.0001
**Error**	33	0.17455	0.00529		
**Corrected Total**	36	0.38887			

**Root MSE**	0.07273	**R-Square**	0.5511
**Dependent Mean**	0.89848	**Adj R-Sq**	0.5103
**Coeff Var**	8.09463		

Parameter Estimates					
**Variable**	**DF**	**Parameter Estimate**	**Standard Error**	**t Value**	**Pr > |t|**
**Intercept**	1	0.92302	0.02539	36.36	<.0001
**Q**	1	-0.00410	0.00074463	-5.50	<.0001
**R166dummy**	1	0.12604	0.03040	4.15	0.0002
**R172dummy**	1	0.11477	0.03021	3.80	0.0006

The SAS System

The REG Procedure
Model: MODEL1
Dependent Variable: HR_norm

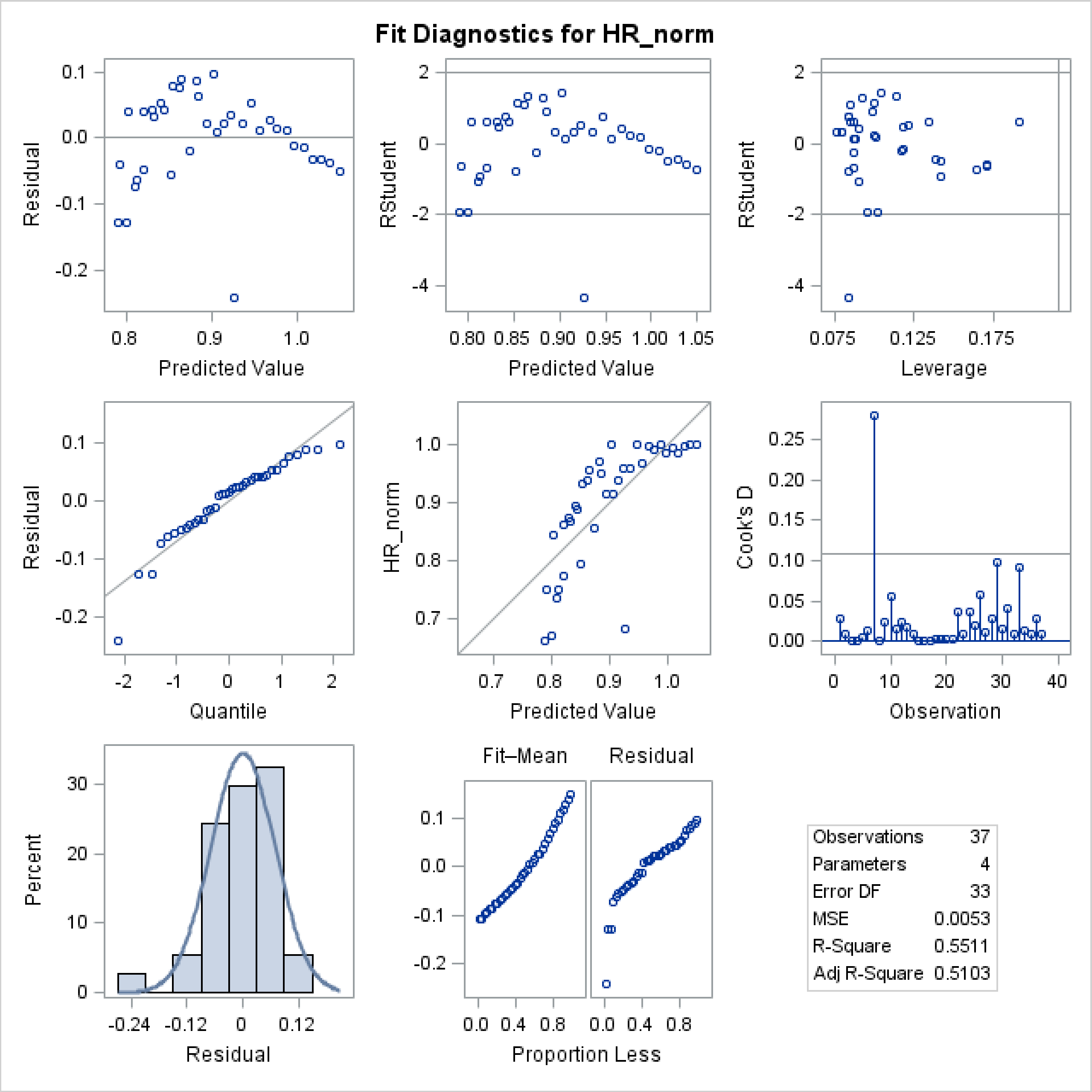

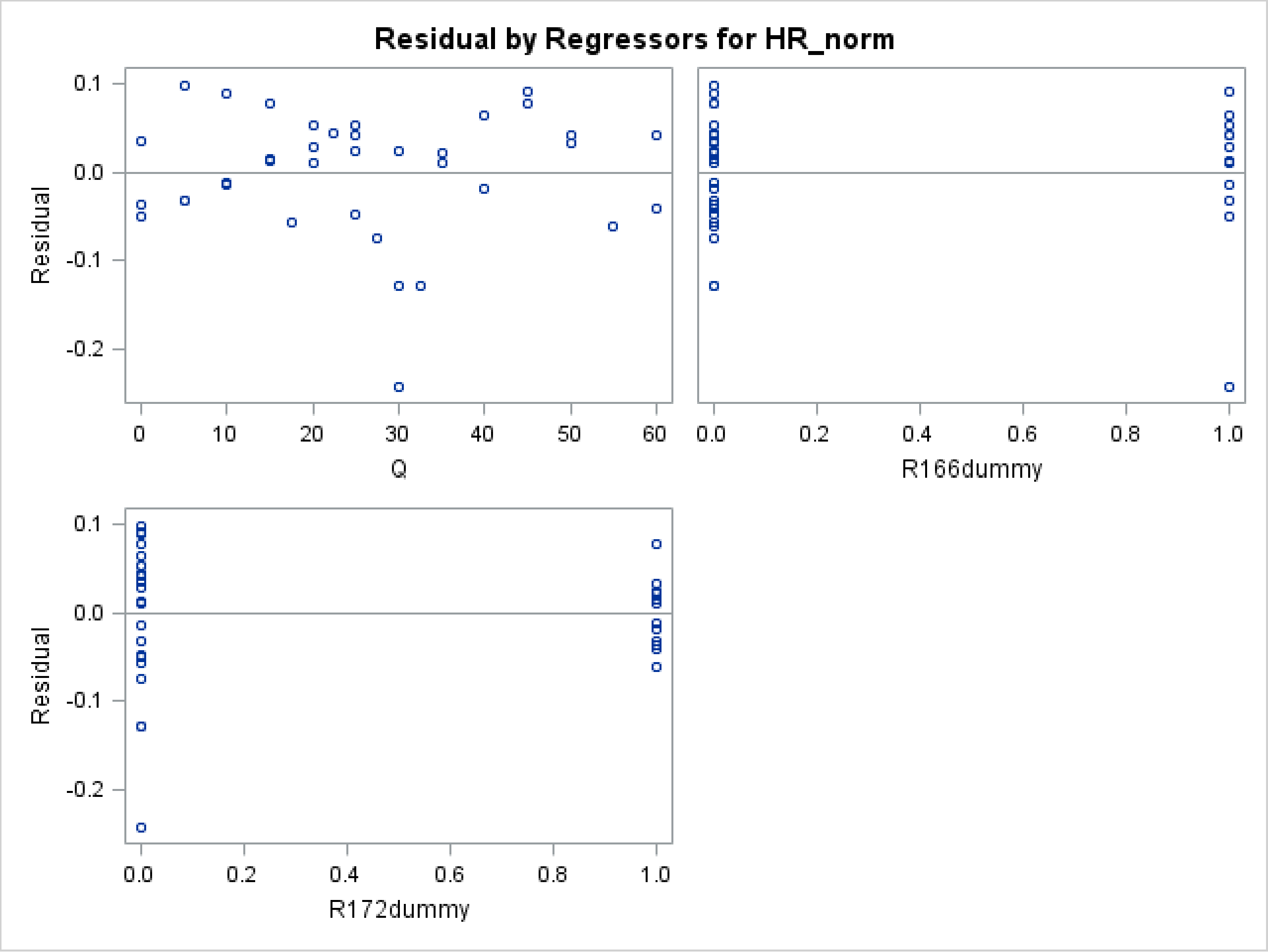
Residual by Regressors for HR_norm
Residual
Q
R166dummy
R172dummy
0.1
0.0
-0.1
-0.2
0
10
20
30
40
50
60
0.0
0.2
0.4
0.6
0.8
1.0

The SAS System

The REG Procedure
Model: MODEL1
Dependent Variable: HR_norm

**Number of Observations Read**	37
**Number of Observations Used**	37

Analysis of Variance

Source	DF	Sum of Squares	Mean Square	F Value	Pr > F
**Model**	3	0.07390	0.02463	2.58	0.0701
**Error**	33	0.31498	0.00954		
**Corrected Total**	36	0.38887			

**Root MSE**	0.09770	**R-Square**	0.1900
**Dependent Mean**	0.89848	**Adj R-Sq**	0.1164
**Coeff Var**	10.87359		

Parameter Estimates

Variable	DF	Parameter Estimate	Standard Error	t Value	Pr > |t|
**Intercept**	1	0.90114	0.04848	18.59	<.0001
**A**	1	-0.07678	0.05345	-1.44	0.1603
**R166dummy**	1	0.08355	0.04015	2.08	0.0453
**R172dummy**	1	0.05337	0.04086	1.31	0.2006

The SAS System

The REG Procedure
Model: MODEL1
Dependent Variable: HR_norm

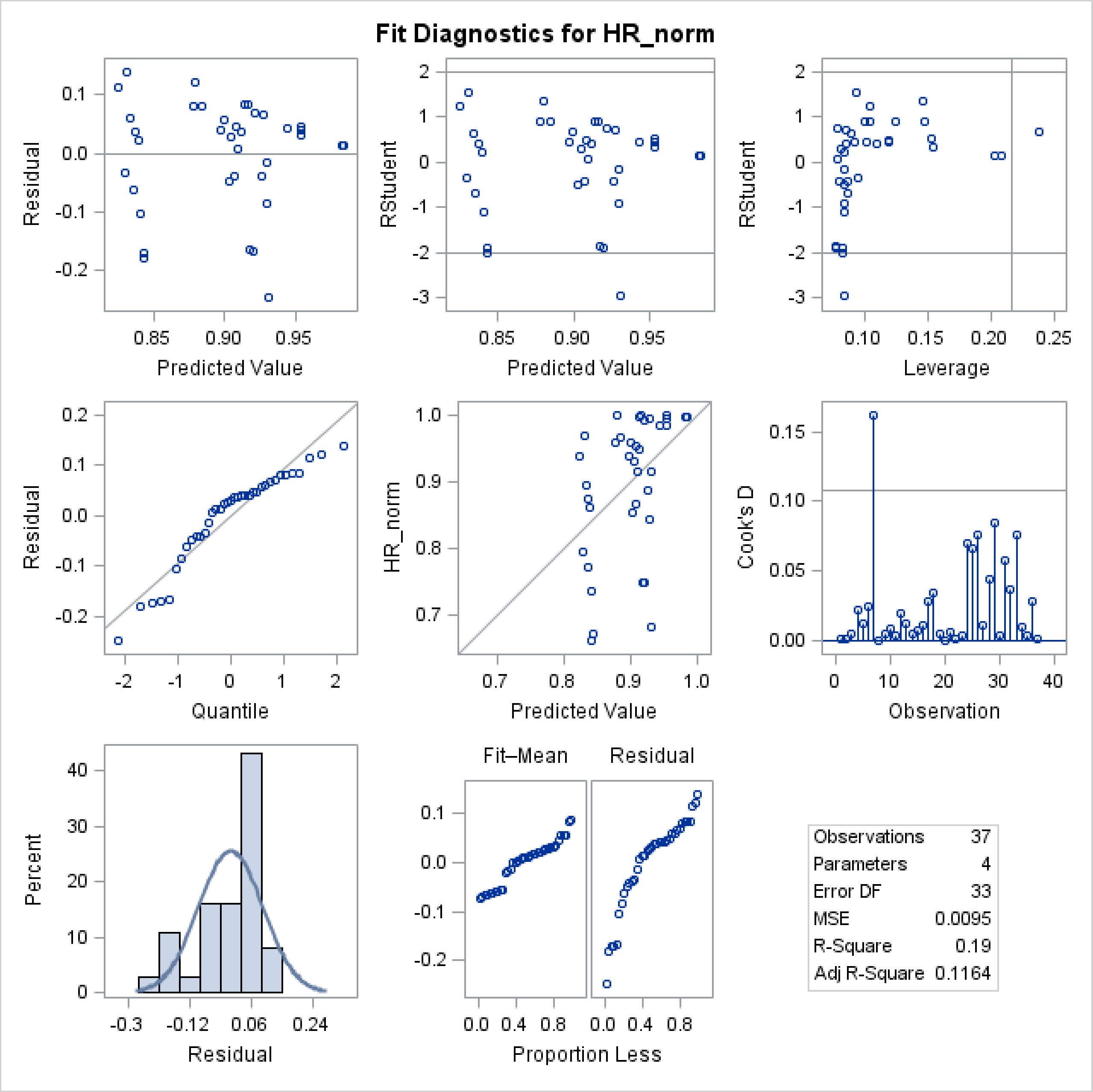

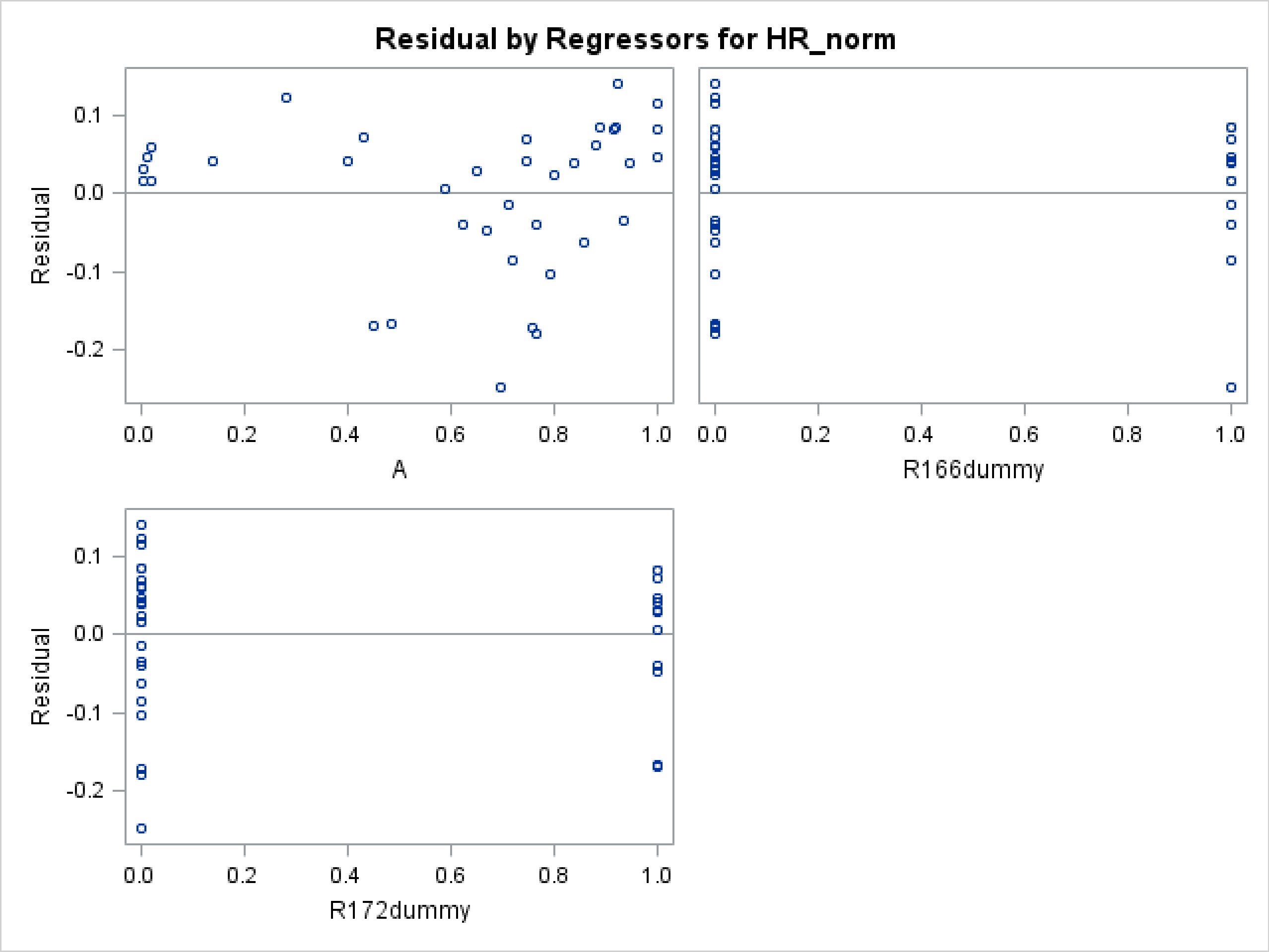
Residual by Regressors for HR_norm
Residual
0.1
0.0
-0.1
-0.2
0.0
0.2
0.4
0.6
0.8
1.0
A
R166dummy
R172dummy

The SAS System

The REG Procedure
Model: MODEL1
Dependent Variable: HR_norm

Number of Observations Read	37
Number of Observations Used	37

Analysis of Variance					
Source	DF	Sum of Squares	Mean Square	F Value	Pr > F
Model	3	0.29007	0.09669	32.29	<.0001
Error	33	0.09881	0.00299		
Corrected Total	36	0.38887			

Root MSE	0.05472	R-Square	0.7459
Dependent Mean	0.89848	Adj R-Sq	0.7228
Coeff Var	6.09011		

Parameter Estimates					
Variable	DF	Parameter Estimate	Standard Error	t Value	Pr > |t|
Intercept	1	0.93421	0.01875	49.82	<.0001
B	1	-0.23462	0.02643	-8.88	<.0001
R166dummy	1	0.08944	0.02234	4.00	0.0003
R172dummy	1	0.06455	0.02191	2.95	0.0059

The SAS System

The REG Procedure
Model: MODEL1
Dependent Variable: HR_norm

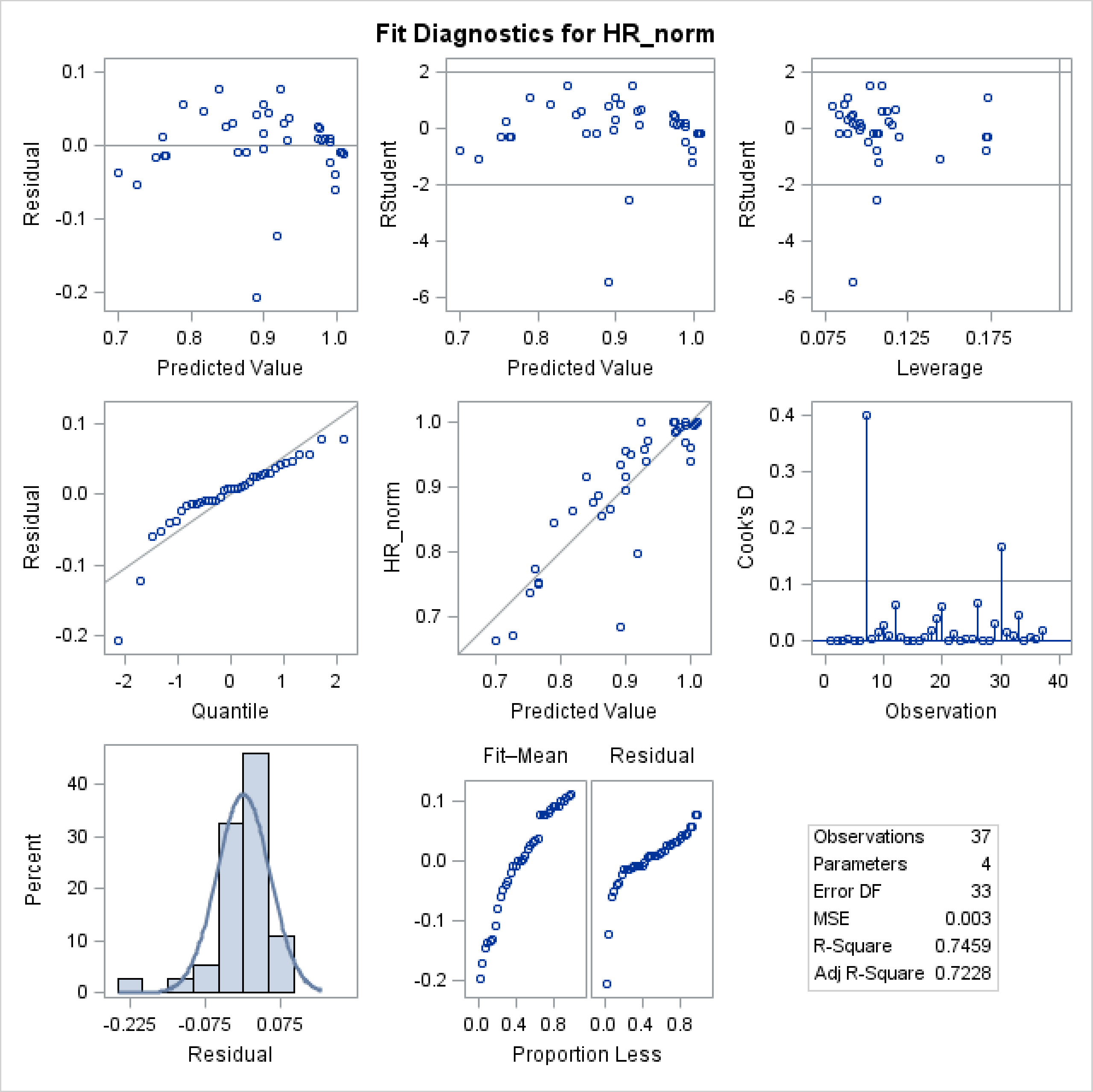

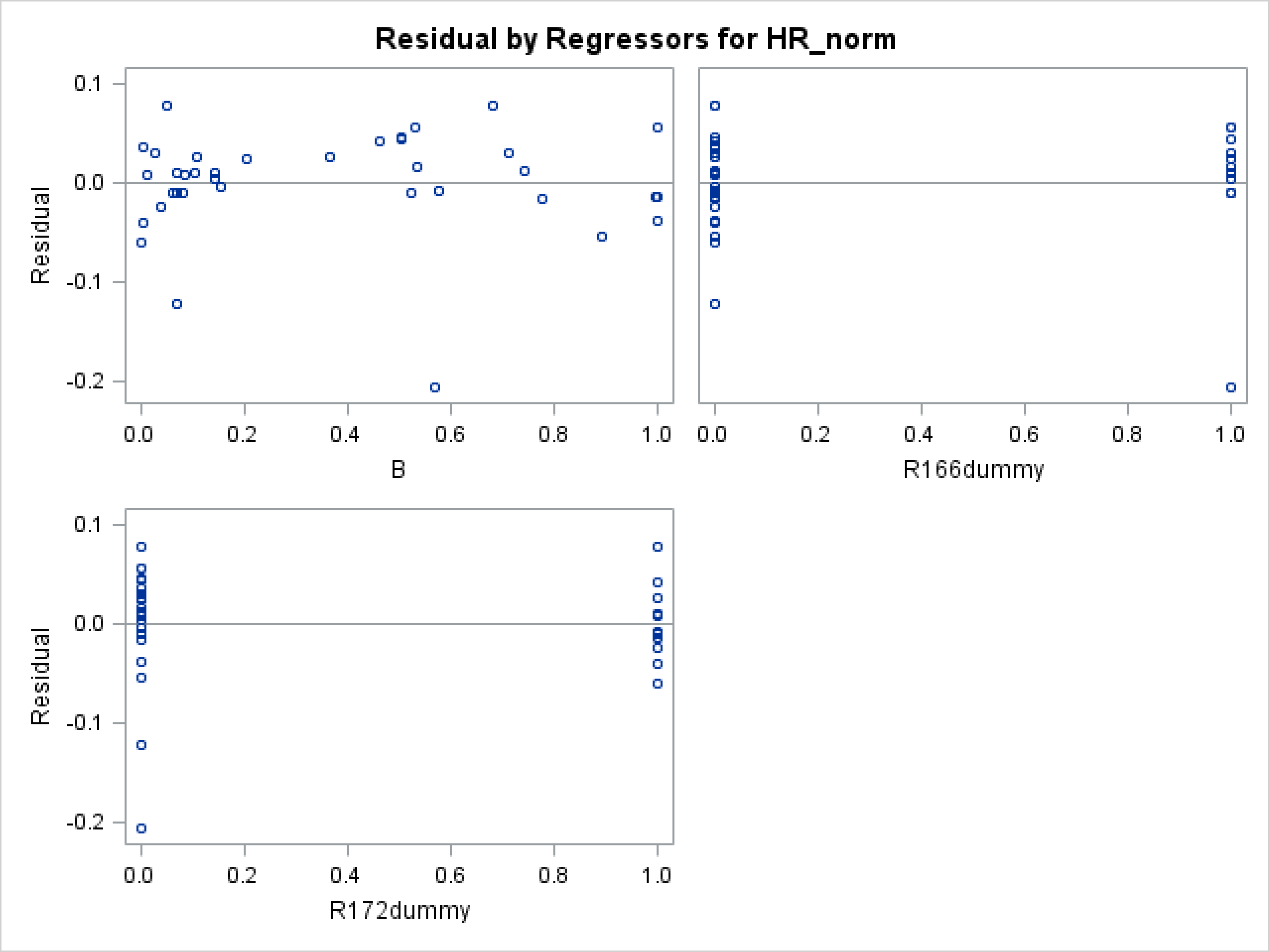
Residual by Regressors for HR_norm
Residual
0.1
0.0
-0.1
-0.2
0.0
0.2
0.4
0.6
0.8
1.0
B
R166dummy
R172dummy

The SAS System

The REG Procedure
Model: MODEL1
Dependent Variable: HR_norm

**Number of Observations Read**	37
**Number of Observations Used**	37

Analysis of Variance					
**Source**	**DF**	**Sum of Squares**	**Mean Square**	**F Value**	**Pr > F**
**Model**	3	0.15790	0.05263	7.52	0.0006
**Error**	33	0.23098	0.00700		
**Corrected Total**	36	0.38887			

**Root MSE**	0.08366	**R-Square**	0.4060
**Dependent Mean**	0.89848	**Adj R-Sq**	0.3520
**Coeff Var**	9.31143		

Parameter Estimates					
**Variable**	**DF**	**Parameter Estimate**	**Standard Error**	**t Value**	**Pr > |t|**
**Intercept**	1	0.98367	0.04348	22.62	<.0001
**C**	1	-0.17545	0.04558	-3.85	0.0005
**R166dummy**	1	0.06211	0.03493	1.78	0.0846
**R172dummy**	1	0.00921	0.03707	0.25	0.8053

The SAS System

The REG Procedure
Model: MODEL1
Dependent Variable: HR_norm

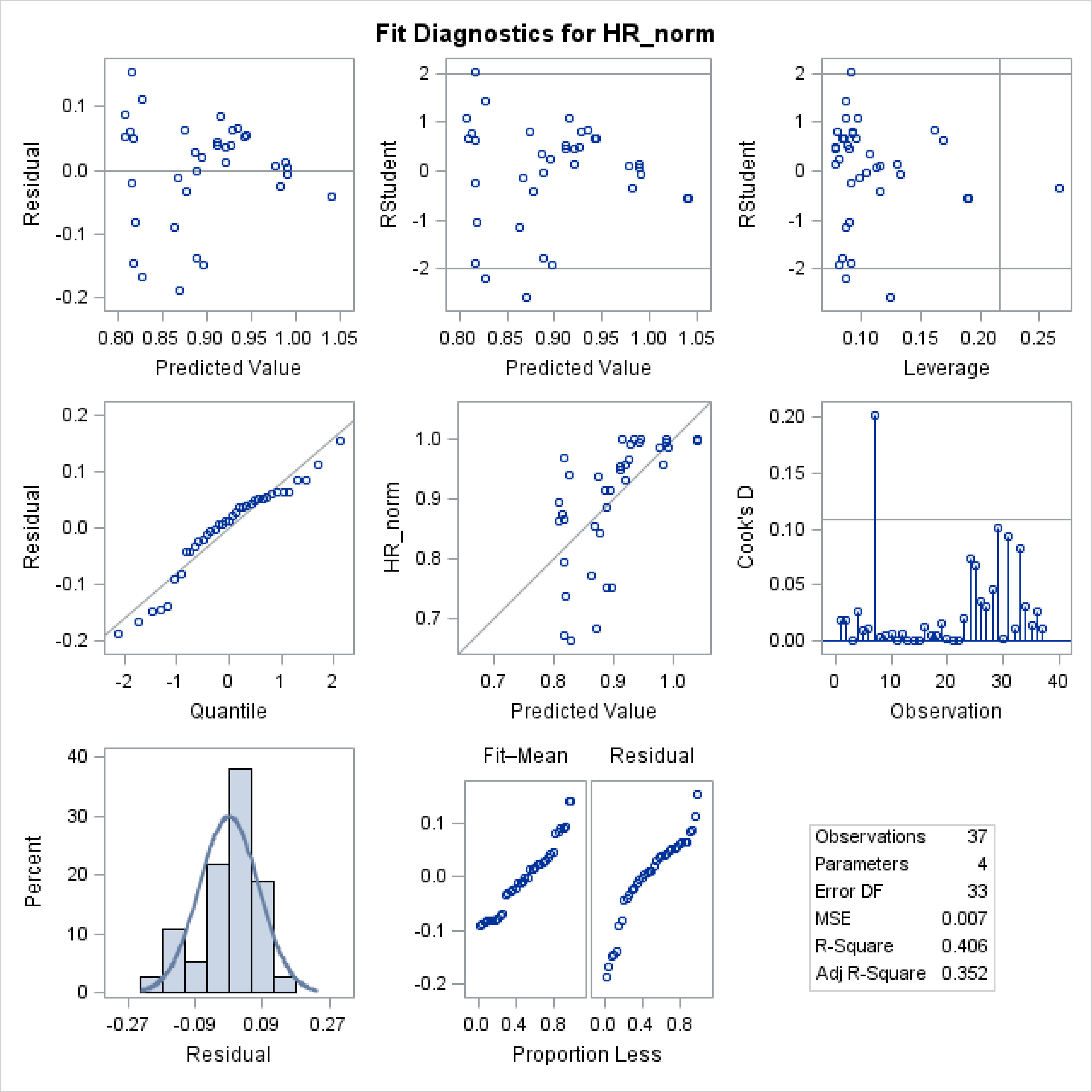

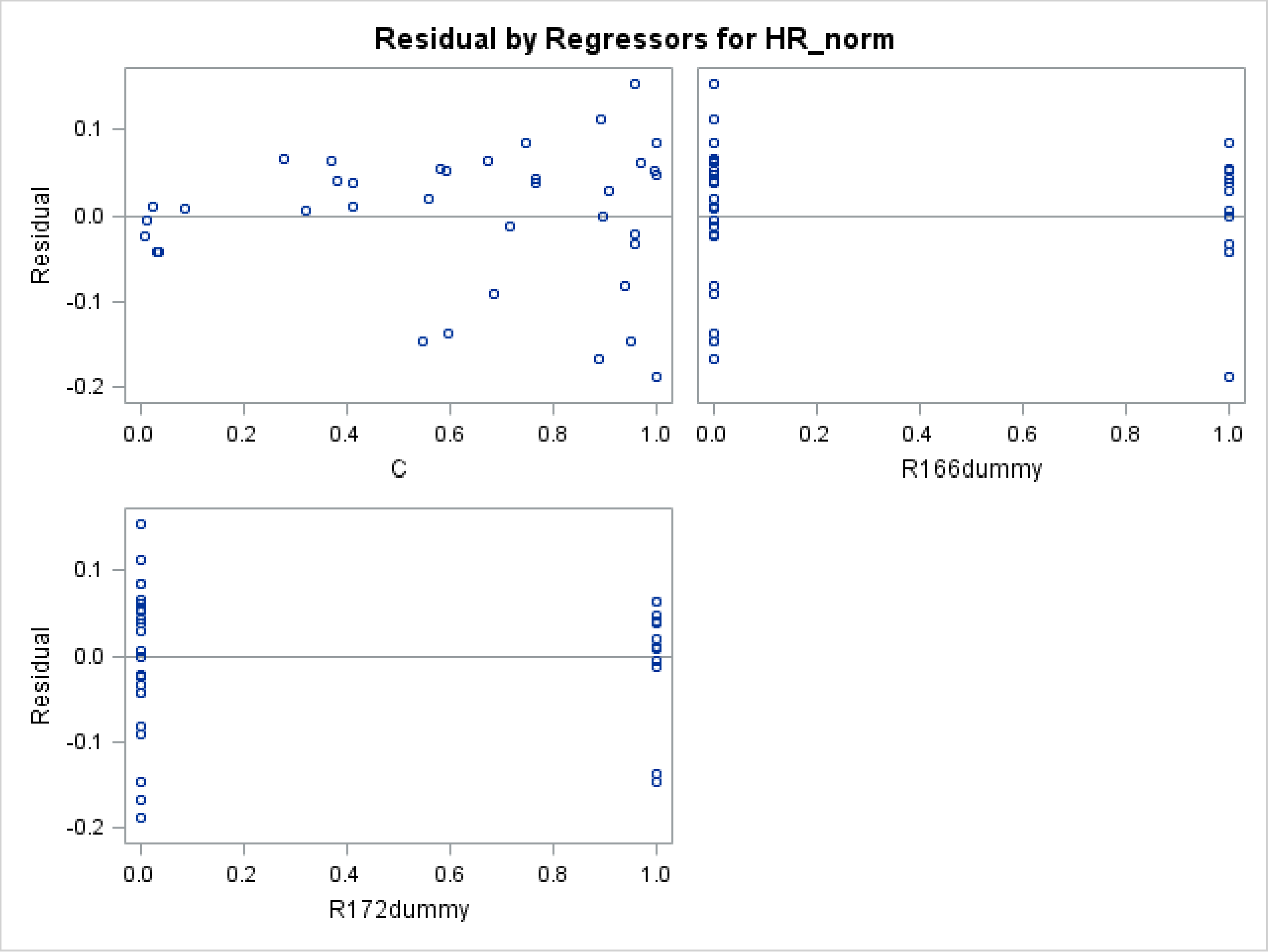

Residual by Regressors for HR_norm
Residual
C
R166dummy
R172dummy
0.1
0.0
-0.1
-0.2
0.0 0.2 0.4 0.6 0.8 1.0

The SAS System

The REG Procedure
Model: MODEL1
Dependent Variable: SV

Number of Observations Read	37
Number of Observations Used	37

Analysis of Variance

Source	DF	Sum of Squares	Mean Square	F Value	Pr > F
Model	3	7408.24373	2469.41458	30.84	<.0001
Error	33	2642.25972	80.06848		
Corrected Total	36	10051			

Root MSE	8.94810	R-Square	0.7371
Dependent Mean	141.10553	Adj R-Sq	0.7132
Coeff Var	6.34142		

Parameter Estimates

Variable	DF	Parameter Estimate	Standard Error	t Value	Pr > |t|
Intercept	1	161.18380	3.06664	52.56	<.0001
B	1	-4.30763	4.32282	-1.00	0.3263
R166dummy	1	-34.78463	3.65307	-9.52	<.0001
R172dummy	1	-20.46910	3.58372	-5.71	<.0001

The SAS System

The REG Procedure
Model: MODEL1
Dependent Variable: SV

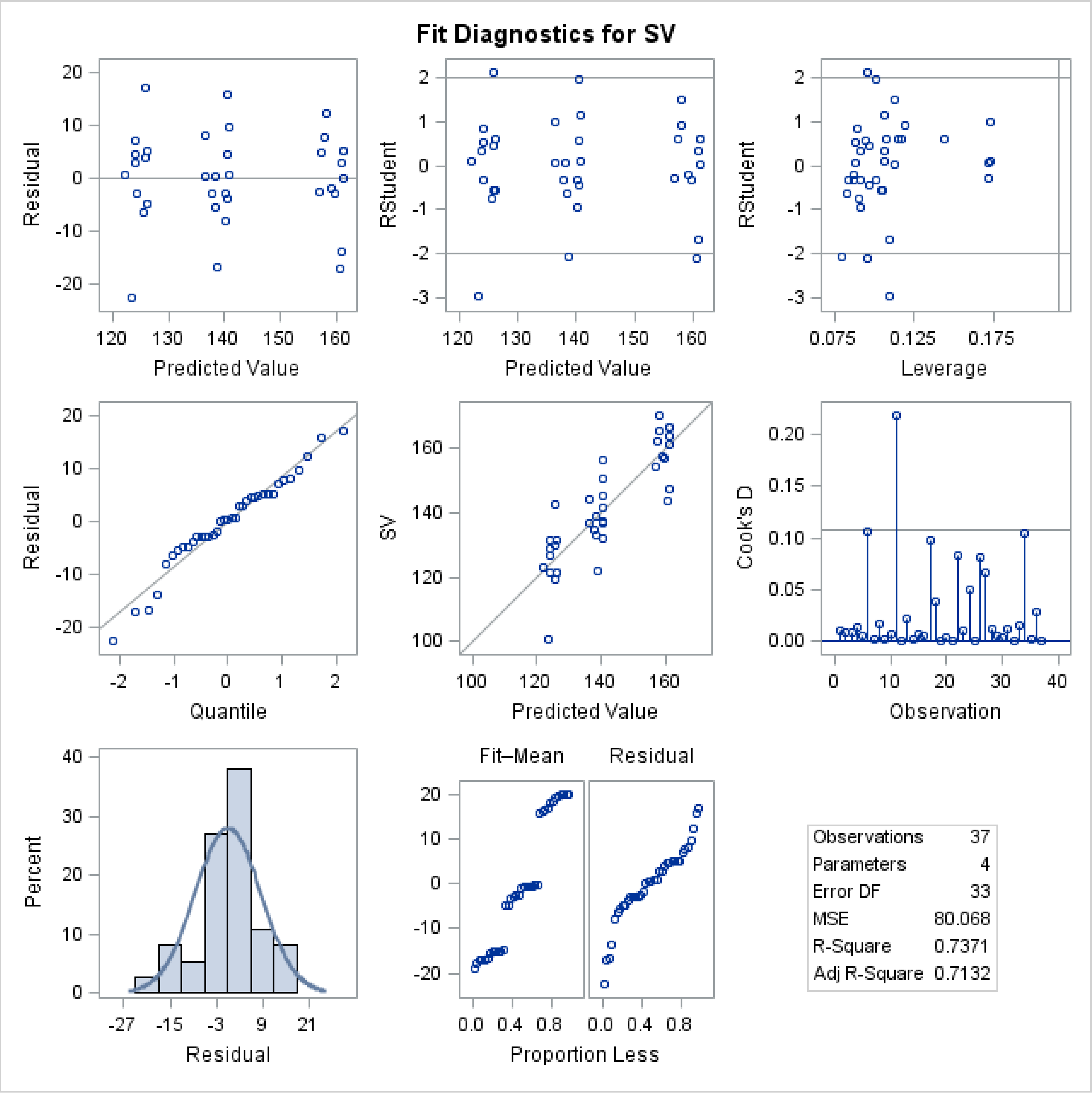

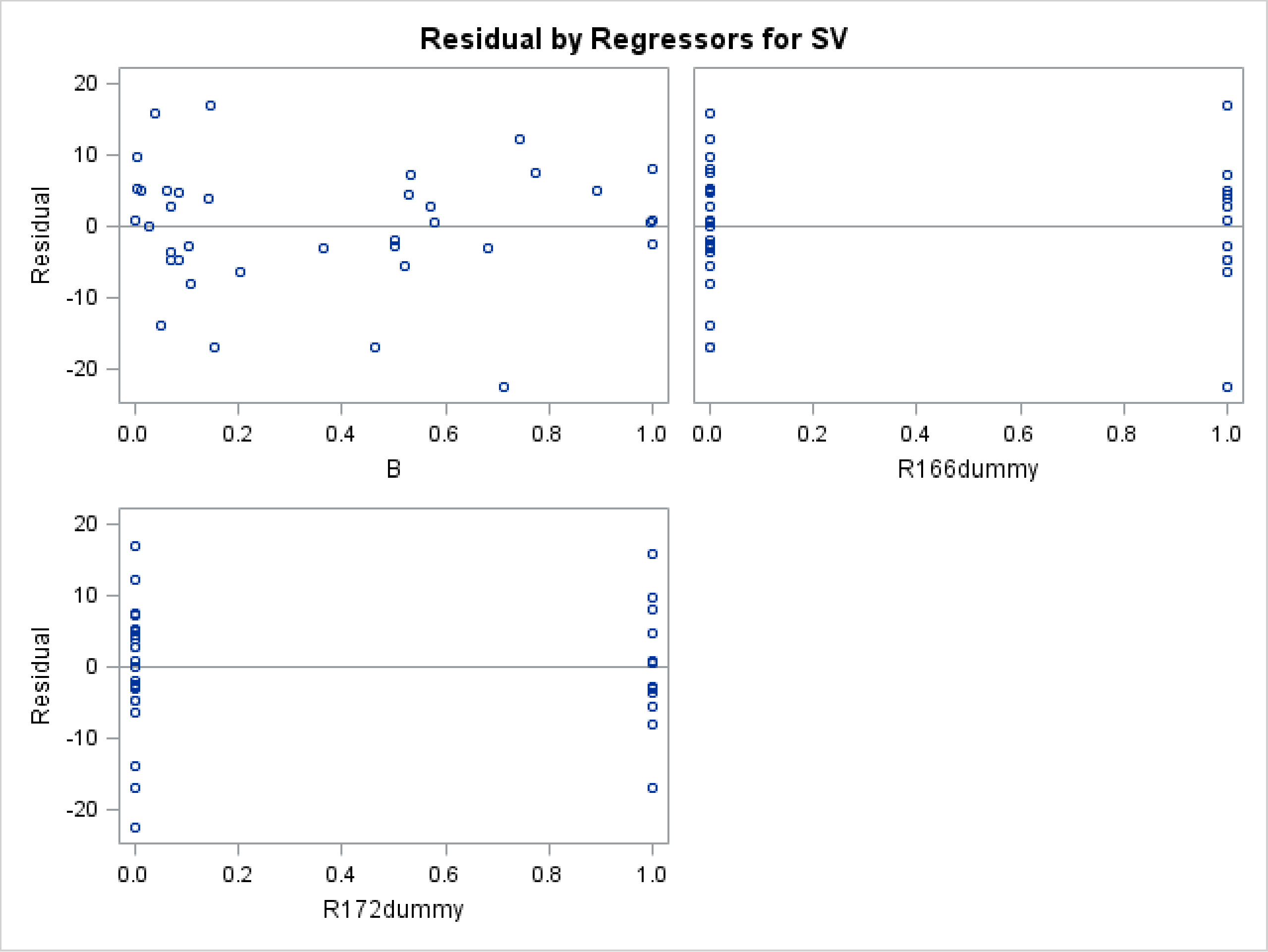

Residual by Regressors for SV
Residual
B
R166dummy
R172dummy
20
10
0
-10
-20
0.0
0.2
0.4
0.6
0.8
1.0

The SAS System

The REG Procedure
Model: MODEL1
Dependent Variable: EF

Number of Observations Read	37
Number of Observations Used	37

Analysis of Variance					
Source	DF	Sum of Squares	Mean Square	F Value	Pr > F
Model	3	1647.71635	549.23878	84.00	<.0001
Error	33	215.78445	6.53892		
Corrected Total	36	1863.50079			

Root MSE	2.55713	R-Square	0.8842
Dependent Mean	74.84876	Adj R-Sq	0.8737
Coeff Var	3.41640		

Parameter Estimates					
Variable	DF	Parameter Estimate	Standard Error	t Value	Pr > |t|
Intercept	1	80.29170	0.87637	91.62	<.0001
B	1	-6.50842	1.23535	-5.27	<.0001
R166dummy	1	-11.93133	1.04395	-11.43	<.0001
R172dummy	1	2.42365	1.02413	2.37	0.0240

The SAS System

The REG Procedure
Model: MODEL1
Dependent Variable: EF

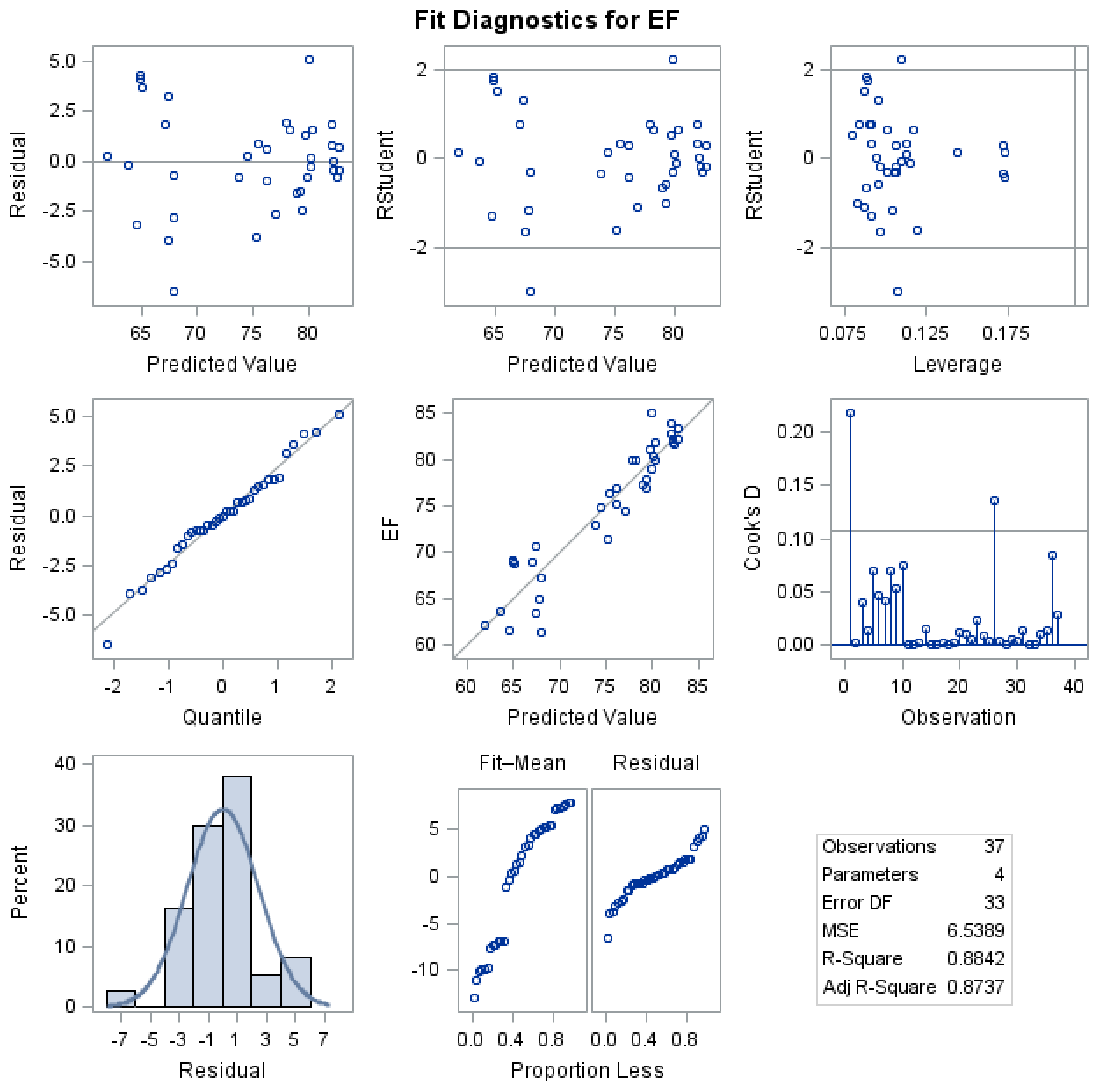

Observations	37
Parameters	4
Error DF	33
MSE	6.5389
R-Square	0.8842
Adj R-Square	0.8737

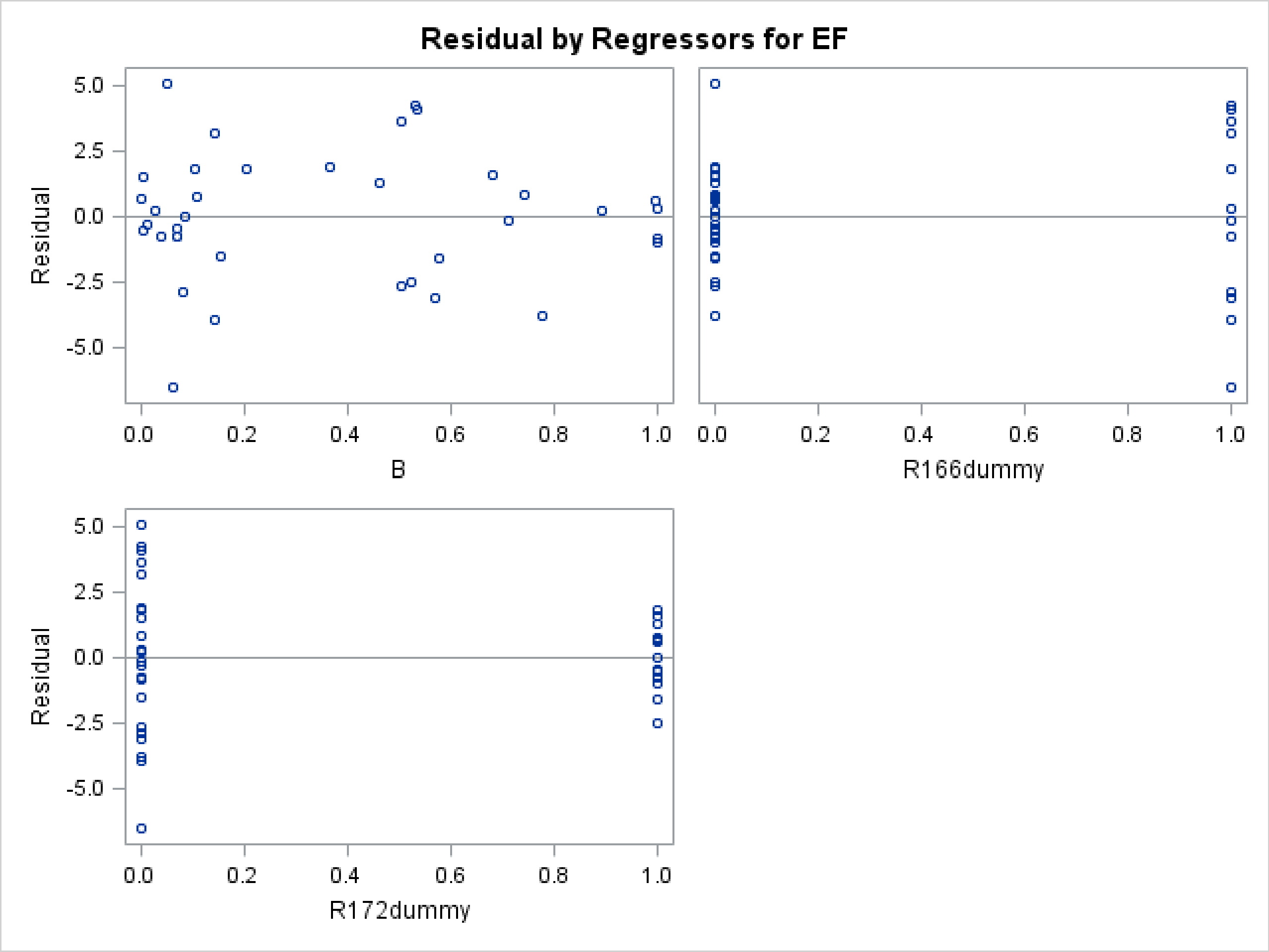

Residual by Regressors for EF
Residual
5.0
2.5
0.0
-2.5
-5.0
0.0
0.2
0.4
0.6
0.8
1.0
B
R166dummy
R172dummy

The SAS System

The REG Procedure
Model: MODEL1
Dependent Variable: FS

Number of Observations Read	37
Number of Observations Used	37

Analysis of Variance					
Source	DF	Sum of Squares	Mean Square	F Value	Pr > F
Model	3	1291.96054	430.65351	86.81	<.0001
Error	33	163.70562	4.96078		
Corrected Total	36	1455.66616			

Root MSE	2.22728	R-Square	0.8875
Dependent Mean	45.03932	Adj R-Sq	0.8773
Coeff Var	4.94519		

Parameter Estimates					
Variable	DF	Parameter Estimate	Standard Error	t Value	Pr > |t|
Intercept	1	50.02341	0.76332	65.53	<.0001
B	1	-6.33329	1.07600	-5.89	<.0001
R166dummy	1	-10.40718	0.90929	-11.45	<.0001
R172dummy	1	2.13703	0.89203	2.40	0.0224

The SAS System

The REG Procedure
Model: MODEL1
Dependent Variable: FS

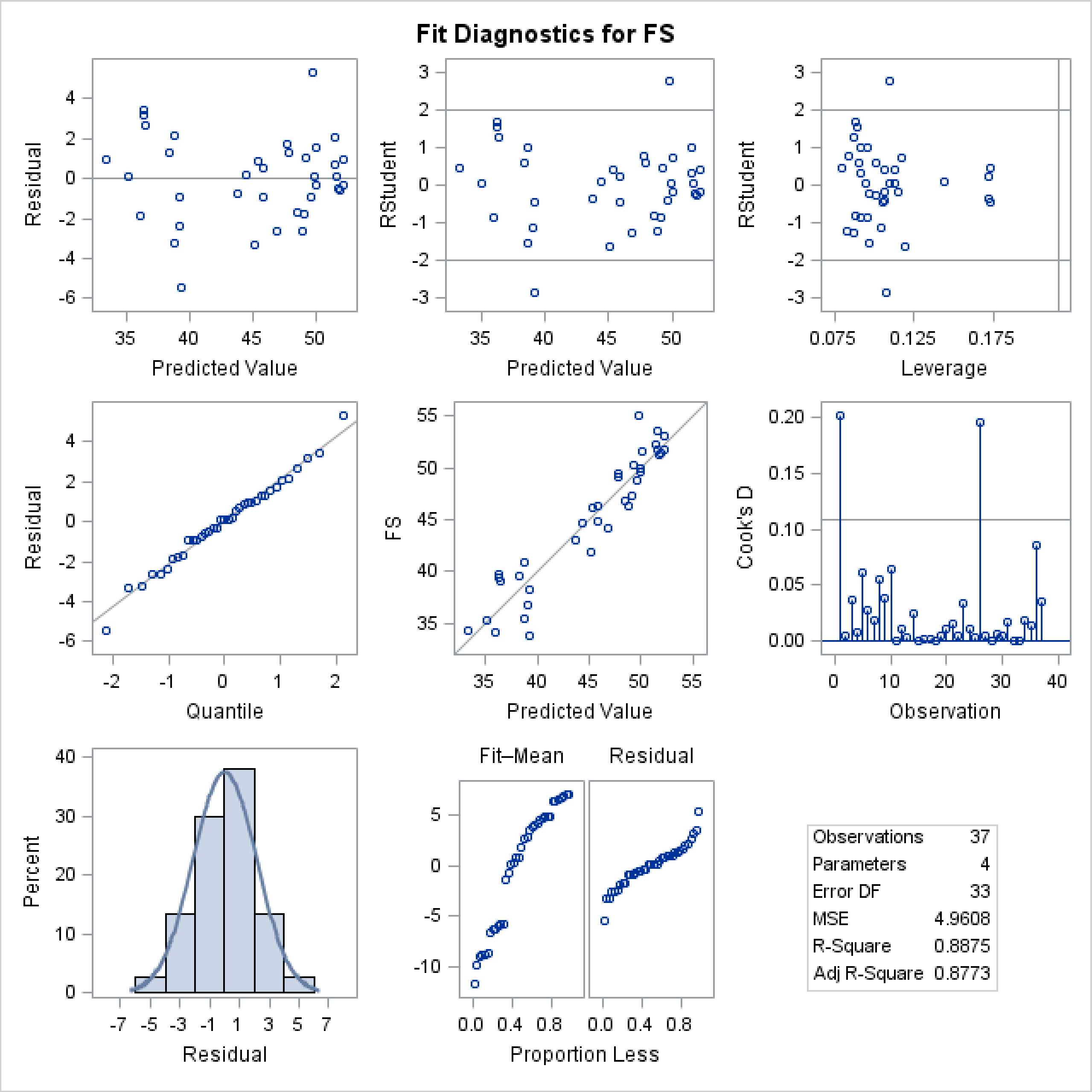

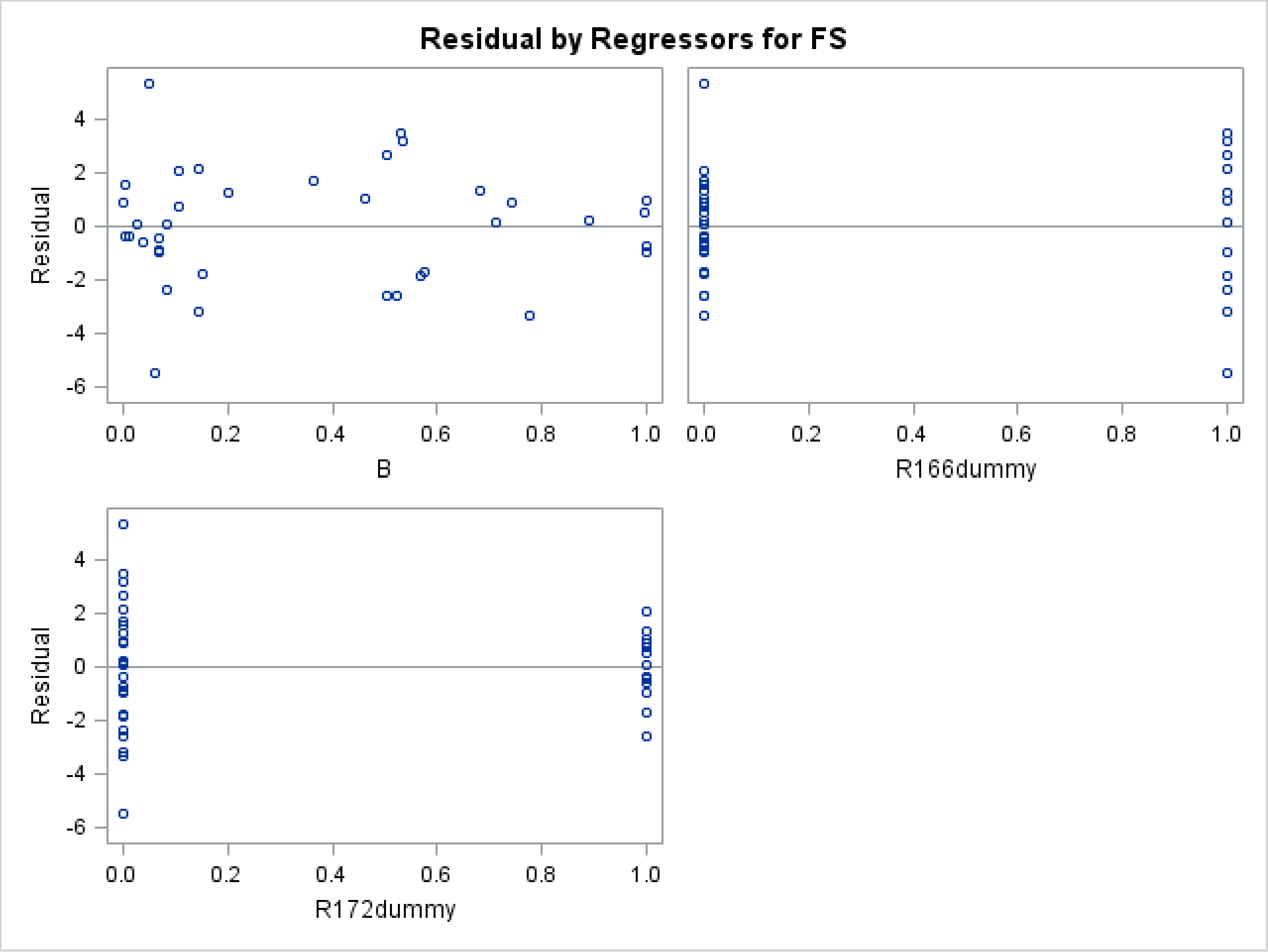

Residual by Regressors for FS
Residual
B
R166dummy
R172dummy
4
2
0
-2
-4
-6
0.0
0.2
0.4
0.6
0.8
1.0

The SAS System

The REG Procedure
Model: MODEL1
Dependent Variable: CO

Number of Observations Read	37
Number of Observations Used	37

Analysis of Variance

Source	DF	Sum of Squares	Mean Square	F Value	Pr > F
Model	3	1314.07639	438.02546	35.42	<.0001
Error	33	408.06249	12.36553		
Corrected Total	36	1722.13888			

Root MSE	3.51647	R-Square	0.7630
Dependent Mean	47.02526	Adj R-Sq	0.7415
Coeff Var	7.47782		

Parameter Estimates

Variable	DF	Parameter Estimate	Standard Error	t Value	Pr > |t|
Intercept	1	55.14066	1.20514	45.75	<.0001
B	1	-14.25802	1.69880	-8.39	<.0001
R166dummy	1	-7.69469	1.43560	-5.36	<.0001
R172dummy	1	-0.87558	1.40835	-0.62	0.5384

The SAS System

The REG Procedure
Model: MODEL1
Dependent Variable: CO

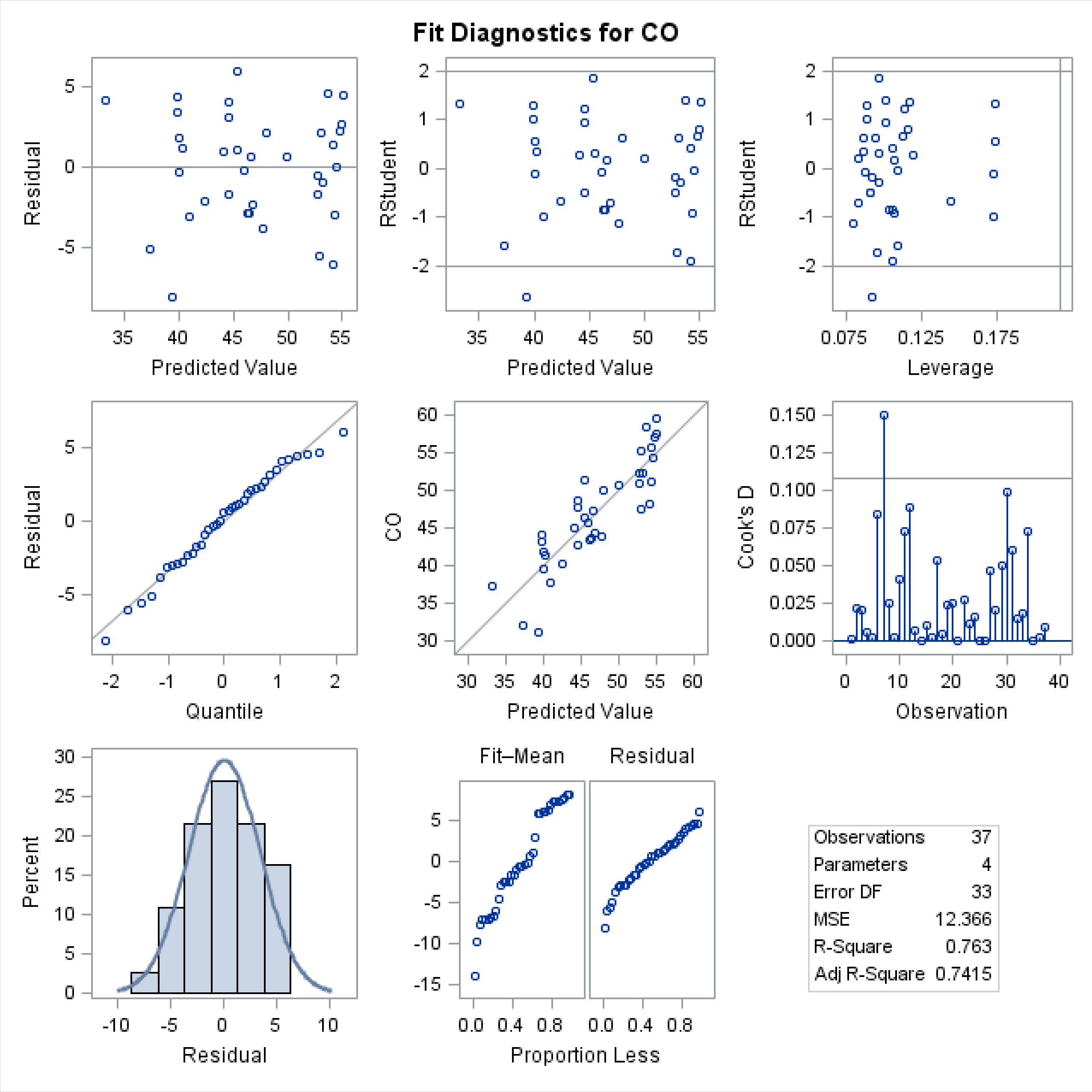

Observations	37
Parameters	4
Error DF	33
MSE	12.366
R-Square	0.763
Adj R-Square	0.7415

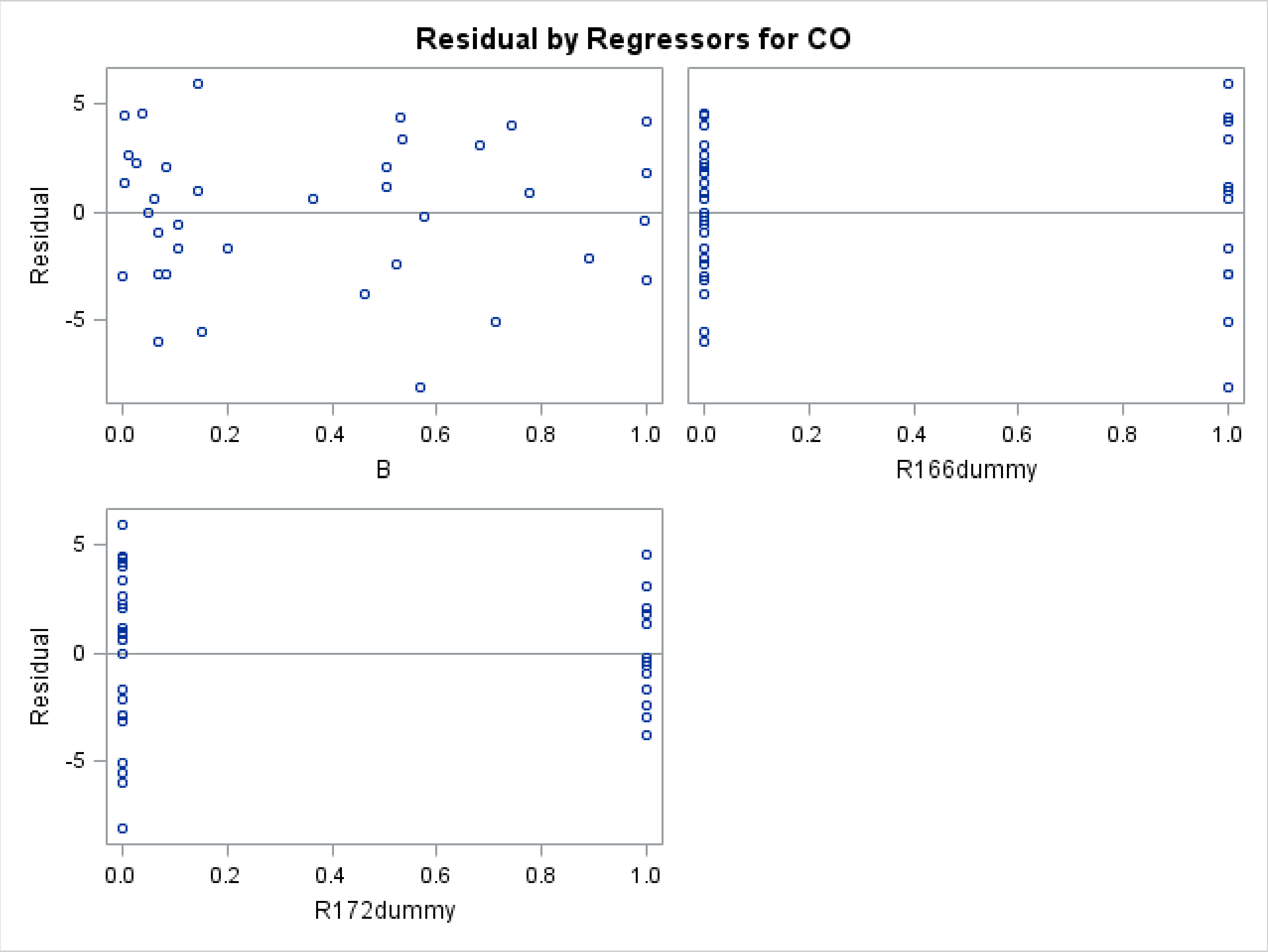
Residual by Regressors for CO
Residual
B
R166dummy
R172dummy
5
0
-5
0.0
0.2
0.4
0.6
0.8
1.0
