# Supplementary material for: B fibers are the best predictors of cardiac activity during Vagus nerve stimulation: Qing, vagal B fiber activation and cardiac effects
Source: Bioelectron Med. 2018 Apr 20;4:5. doi: 10.1186/s42234-018-0005-8 (PMC7098216; doi:10.1186/s42234-018-0005-8)
Supplement: Supplementary file 4 — Results from regression analysis of heart rate and sonographic data using SAS. (PDF 412 kb) [file 42234_2018_5_MOESM4_ESM.pdf]

The SAS System

Obs	Rat	Q	A	B	C	HR_norm	R153dummy	R154dummy	R162dummy	R163dummy	R164dummy
1	R153	0.0	0.03757	0.02625	0.04517	0.96987	1	0	0	0	0
2	R153	6.0	0.24665	0.14945	0.27477	1.00000	1	0	0	0	0
3	R153	9.0	0.29311	0.19160	0.37670	0.94332	1	0	0	0	0
4	R153	12.0	0.51534	0.46254	0.35317	0.88481	1	0	0	0	0
5	R153	15.0	0.43986	0.29355	0.47341	0.85460	1	0	0	0	0
6	R153	18.0	0.69452	0.88736	0.09346	0.79223	1	0	0	0	0
7	R153	24.0	0.75878	0.92298	0.13004	0.71566	1	0	0	0	0
8	R153	0.0	0.05334	0.03142	0.04193	0.99655	1	0	0	0	0
9	R153	6.0	0.29398	0.09271	0.21370	0.99012	1	0	0	0	0
10	R153	9.0	0.54440	0.35610	0.21963	0.93384	1	0	0	0	0
11	R153	12.0	0.28928	0.12121	0.38004	0.95727	1	0	0	0	0
12	R153	15.0	0.41323	0.47393	0.11855	0.92732	1	0	0	0	0
13	R153	18.0	0.24988	0.33718	0.42039	0.84855	1	0	0	0	0
14	R153	24.0	0.40475	0.67549	0.31320	0.73576	1	0	0	0	0
15	R153	30.0	0.48592	0.50000	0.52773	0.83669	1	0	0	0	0
16	R153	36.0	0.59371	0.71943	0.33304	0.73576	1	0	0	0	0
17	R153	0.0	0.05298	0.01714	0.05618	0.97062	1	0	0	0	0
18	R153	6.0	0.53903	0.17313	0.04400	0.96245	1	0	0	0	0
19	R153	9.0	0.35994	0.25283	0.17583	0.96054	1	0	0	0	0
20	R153	12.0	0.33631	0.19393	0.44242	0.95304	1	0	0	0	0
21	R153	15.0	0.23098	0.11701	0.42540	0.97125	1	0	0	0	0
22	R153	18.0	0.46822	0.43880	0.37414	0.82185	1	0	0	0	0
23	R153	24.0	0.27254	0.13504	0.73672	0.90871	1	0	0	0	0
24	R153	30.0	0.39736	0.33347	0.61020	0.75452	1	0	0	0	0
25	R153	0.0	0.06224	0.05580	0.01992	0.99203	1	0	0	0	0
26	R153	6.0	0.20649	0.16780	0.08171	0.98088	1	0	0	0	0
27	R153	9.0	0.37261	0.31729	0.11804	0.97094	1	0	0	0	0
28	R153	12.0	0.33209	0.17494	0.26786	0.95544	1	0	0	0	0
29	R153	15.0	0.14273	0.16856	0.44608	0.97021	1	0	0	0	0
30	R153	18.0	0.11983	0.09746	0.46421	0.97818	1	0	0	0	0
31	R153	24.0	0.18858	0.15246	0.59000	0.96781	1	0	0	0	0
32	R153	30.0	0.58206	1.00000	-0.00240	0.79237	1	0	0	0	0
33	R153	36.0	0.58305	0.68897	0.36093	0.77419	1	0	0	0	0
34	R153	0.0	0.04014	0.01665	0.00606	0.95294	1	0	0	0	0
35	R153	6.0	0.28222	0.05432	0.14078	0.96540	1	0	0	0	0
36	R153	12.0	0.29567	0.11185	0.32917	0.93421	1	0	0	0	0
37	R153	18.0	0.26022	0.12755	0.68608	0.91831	1	0	0	0	0
38	R153	24.0	1.00000	0.50322	0.46372	0.82809	1	0	0	0	0
39	R153	0.0	0.03750	0.03276	0.04681	0.97895	1	0	0	0	0
40	R153	6.0	0.28592	0.04086	0.03174	0.95372	1	0	0	0	0
41	R153	15.0	0.28180	0.20075	0.34458	0.91139	1	0	0	0	0
42	R153	18.0	0.31729	0.10658	0.54132	0.91350	1	0	0	0	0
43	R153	24.0	0.23385	0.19933	0.71136	0.88540	1	0	0	0	0
44	R153	30.0	0.14187	0.21418	1.00000	0.88006	1	0	0	0	0

45	R153	0.0	-0.00068	0.03404	0.07633	0.95875	1	0	0	0	0
46	R153	6.0	0.25302	0.03099	0.04174	0.95836	1	0	0	0	0
47	R153	9.0	0.38130	0.01306	0.12071	0.95411	1	0	0	0	0
48	R153	12.0	0.32864	0.13923	0.24437	0.93465	1	0	0	0	0
49	R153	15.0	0.27973	0.13260	0.33830	0.91822	1	0	0	0	0
50	R153	24.0	0.17444	0.19385	0.48416	0.90192	1	0	0	0	0
51	R153	30.0	0.11505	0.20866	0.94883	0.88362	1	0	0	0	0
52	R153	0.0	0.05327	0.04187	0.04155	0.96805	1	0	0	0	0
53	R153	6.0	0.06066	0.03209	0.05511	0.98634	1	0	0	0	0
54	R153	9.0	0.19083	0.01538	0.09971	0.95838	1	0	0	0	0
55	R153	12.0	0.18506	0.09036	0.34179	0.96502	1	0	0	0	0
56	R153	15.0	0.11546	0.09501	0.27969	0.91734	1	0	0	0	0
57	R154	26.4	0.49864	0.34230	0.67913	0.97234	0	1	0	0	0
58	R154	70.4	0.66160	0.81155	0.75232	0.84748	0	1	0	0	0
59	R154	35.2	0.36012	0.28995	0.72189	0.97463	0	1	0	0	0
60	R154	17.6	0.16642	0.10595	0.54295	0.98830	0	1	0	0	0
61	R154	70.4	0.61117	0.96671	0.78127	0.78909	0	1	0	0	0
62	R154	26.4	0.51430	0.39925	0.64668	0.96635	0	1	0	0	0
63	R154	52.8	0.58058	0.51022	0.76090	0.91439	0	1	0	0	0
64	R154	0.0	0.01752	0.01295	0.03099	0.97122	0	1	0	0	0
65	R154	105.6	0.53974	0.97980	0.71814	0.78100	0	1	0	0	0
66	R154	17.6	0.24219	0.18328	0.69829	0.95951	0	1	0	0	0
67	R154	17.6	0.18442	0.18451	0.70408	0.96048	0	1	0	0	0
68	R154	105.6	0.53425	1.00000	0.82476	0.82293	0	1	0	0	0
69	R154	52.8	0.48918	0.59136	0.77132	0.83221	0	1	0	0	0
70	R154	0.0	0.00754	0.00998	0.01625	0.92127	0	1	0	0	0
71	R154	0.0	0.01162	0.00538	0.02703	0.92165	0	1	0	0	0
72	R154	44.0	0.33080	0.30836	0.61221	0.92449	0	1	0	0	0
73	R154	35.2	0.40840	0.44953	0.66973	0.88479	0	1	0	0	0
74	R154	88.0	0.45725	0.82179	0.70757	0.67615	0	1	0	0	0
75	R154	52.8	0.36526	0.34193	0.67094	0.91218	0	1	0	0	0
76	R154	70.4	0.45813	0.43813	0.74066	0.84511	0	1	0	0	0
77	R154	44.0	0.40679	0.41664	0.82706	0.87363	0	1	0	0	0
78	R154	105.6	1.00000	0.86391	0.92830	0.73004	0	1	0	0	0
79	R154	44.0	0.41486	0.72776	0.91958	0.77061	0	1	0	0	0
80	R154	26.4	0.10941	0.24504	0.86561	0.89974	0	1	0	0	0
81	R154	35.2	0.27185	0.49711	0.90118	0.90432	0	1	0	0	0
82	R154	32.0	0.46651	0.62015	0.98646	0.79396	0	1	0	0	0
83	R154	12.0	0.06291	0.08823	0.49786	0.93616	0	1	0	0	0
84	R154	24.0	0.12245	0.16608	0.92167	0.95386	0	1	0	0	0
85	R154	48.0	0.32548	0.41385	0.90966	0.93128	0	1	0	0	0
86	R154	32.0	0.18217	0.20438	0.85831	0.96314	0	1	0	0	0
87	R154	20.0	0.21170	0.35189	0.89655	0.96596	0	1	0	0	0
88	R154	17.6	0.24571	0.39729	1.00000	0.97688	0	1	0	0	0
89	R154	0.0	0.01030	0.01268	0.03637	0.98069	0	1	0	0	0
90	R154	26.4	0.30103	0.41473	0.89551	0.98568	0	1	0	0	0
91											

	R154	52.8	0.39323	0.44456	0.80542	0.97236	0	1	0	0	0
92	R154	10.0	0.33372	0.16968	0.46391	0.98501	0	1	0	0	0
93	R154	15.0	0.24268	0.10263	0.38532	1.00000	0	1	0	0	0
94	R154	50.0	0.75986	0.93102	0.64421	0.87997	0	1	0	0	0
95	R154	60.0	0.55956	0.94403	0.57718	0.77648	0	1	0	0	0
96	R154	0.0	0.00803	0.00567	0.00130	0.94005	0	1	0	0	0
97	R154	20.0	0.53108	0.37001	0.80314	0.89078	0	1	0	0	0
98	R154	25.0	0.39228	0.32125	0.78023	0.92987	0	1	0	0	0
99	R154	0.0	0.01588	0.01184	0.02682	0.92591	0	1	0	0	0
100	R154	60.0	0.40430	0.49208	0.80792	0.90314	0	1	0	0	0
101	R154	30.0	0.46236	0.48296	0.91207	0.90188	0	1	0	0	0
102	R154	15.0	0.32640	0.20241	0.81762	0.92145	0	1	0	0	0
103	R154	30.0	0.21233	0.13769	0.86372	0.92167	0	1	0	0	0
104	R154	40.0	0.44946	0.68466	0.93182	0.82361	0	1	0	0	0
105	R154	25.0	0.43460	0.37639	0.90741	0.90830	0	1	0	0	0
106	R154	15.0	0.27575	0.14218	0.86007	0.92922	0	1	0	0	0
107	R154	30.0	0.48760	0.45900	0.96985	0.91336	0	1	0	0	0
108	R154	30.0	0.23997	0.11409	0.86819	0.93498	0	1	0	0	0
109	R154	0.0	0.01587	0.00988	0.02340	0.98196	0	1	0	0	0
110	R154	20.0	0.34925	0.19603	0.79539	0.96674	0	1	0	0	0
111	R162	21.0	0.43854	0.55899	0.87553	0.90554	0	0	1	0	0
112	R162	15.0	0.28676	0.45759	0.78343	0.97622	0	0	1	0	0
113	R162	9.0	0.05449	0.30583	0.51210	0.97214	0	0	1	0	0
114	R162	27.0	0.53912	0.62440	0.92760	0.87518	0	0	1	0	0
115	R162	0.0	0.01703	0.02390	0.02475	0.92152	0	0	1	0	0
116	R162	0.0	0.00718	0.01455	0.02568	0.87619	0	0	1	0	0
117	R162	6.0	0.02245	0.19783	0.39522	0.94523	0	0	1	0	0
118	R162	12.0	0.18602	0.36597	0.73048	0.78402	0	0	1	0	0
119	R162	24.0	0.82642	0.53768	0.93181	0.89225	0	0	1	0	0
120	R162	36.0	0.57305	0.57538	0.91549	0.93863	0	0	1	0	0
121	R162	18.0	0.44165	0.36044	0.87895	0.89744	0	0	1	0	0
122	R162	30.0	0.72660	0.51183	0.98257	0.81586	0	0	1	0	0
123	R162	12.0	0.20367	0.18502	0.55735	0.83965	0	0	1	0	0
124	R162	24.0	1.00000	0.37903	0.84060	0.90972	0	0	1	0	0
125	R162	18.0	0.49833	0.43329	0.80356	1.00000	0	0	1	0	0
126	R162	36.0	0.59670	0.42850	0.94021	0.94210	0	0	1	0	0
127	R162	30.0	0.74328	0.34704	0.90246	0.81097	0	0	1	0	0
128	R162	6.0	0.00296	0.06211	0.25773	0.85835	0	0	1	0	0
129	R162	9.0	0.00303	0.12662	0.38755	0.93255	0	0	1	0	0
130	R162	15.0	0.40553	0.27202	0.75300	0.94832	0	0	1	0	0
131	R162	0.0	0.00516	0.02116	0.02112	0.81892	0	0	1	0	0
132	R162	30.0	0.00161	0.47339	0.92250	0.93675	0	0	1	0	0
133	R162	6.0	0.04522	0.19591	0.39863	0.82101	0	0	1	0	0
134	R162	15.0	0.35166	0.29627	0.81522	0.87982	0	0	1	0	0
135	R162	9.0	0.17623	0.21466	0.64591	0.96691	0	0	1	0	0
136	R162	0.0	0.01307	0.01379	0.07131	0.91729	0	0	1	0	0
137											

	R162	12.0	0.23209	0.30381	0.79078	0.86826	0	0	1	0	0
138	R162	18.0	0.66731	0.22760	0.82627	0.82590	0	0	1	0	0
139	R162	24.0	-0.01622	0.24687	0.91338	0.80132	0	0	1	0	0
140	R162	42.0	0.38119	0.19150	0.96099	0.94365	0	0	1	0	0
141	R162	20.0	0.07136	0.68356	-0.21781	0.62027	0	0	1	0	0
142	R162	20.0	0.08498	0.73961	0.72411	0.61793	0	0	1	0	0
143	R162	20.0	0.02779	0.76001	0.59721	0.61298	0	0	1	0	0
144	R162	40.0	0.05366	0.64421	0.80757	0.60798	0	0	1	0	0
145	R162	50.0	0.07560	0.68904	0.82864	0.60109	0	0	1	0	0
146	R162	60.0	0.08714	0.63103	0.76900	0.59211	0	0	1	0	0
147	R162	70.0	0.12943	0.69072	0.78491	0.59030	0	0	1	0	0
148	R162	100.0	0.21653	1.00000	0.83650	0.57055	0	0	1	0	0
149	R162	40.0	0.04373	0.66004	0.80809	0.74875	0	0	1	0	0
150	R162	35.0	0.12885	0.71850	0.76935	0.76613	0	0	1	0	0
151	R162	30.0	0.03196	0.74594	0.77062	0.77364	0	0	1	0	0
152	R162	25.0	0.02201	0.79522	0.71812	0.76908	0	0	1	0	0
153	R162	20.0	0.01979	0.60165	0.62459	0.79170	0	0	1	0	0
154	R162	15.0	0.01860	0.40993	0.48940	0.83836	0	0	1	0	0
155	R162	20.0	0.03174	0.84292	0.87165	0.76571	0	0	1	0	0
156	R162	40.0	0.02868	0.78267	1.00000	0.77853	0	0	1	0	0
157	R162	30.0	0.12244	0.78367	0.95814	0.80967	0	0	1	0	0
158	R162	20.0	0.00678	0.77221	0.87782	0.82579	0	0	1	0	0
159	R163	14.4	0.59432	0.07715	0.53774	0.98503	0	0	0	1	0
160	R163	7.2	0.30960	0.10189	0.33115	0.98661	0	0	0	1	0
161	R163	19.2	0.61119	0.03767	0.54308	0.98226	0	0	0	1	0
162	R163	24.0	0.61281	0.23218	0.49909	0.98024	0	0	0	1	0
163	R163	12.0	0.56727	0.11554	0.58360	0.98279	0	0	0	1	0
164	R163	0.0	0.00725	0.01185	0.05411	0.99537	0	0	0	1	0
165	R163	4.8	0.13967	0.03556	0.29026	0.98792	0	0	0	1	0
166	R163	9.6	0.54771	0.15055	0.57931	0.98208	0	0	0	1	0
167	R163	28.8	0.61649	0.68613	0.57586	0.98580	0	0	0	1	0
168	R163	33.6	0.61751	0.80483	0.54531	0.98202	0	0	0	1	0
169	R163	7.2	0.15037	0.03765	0.31778	0.98934	0	0	0	1	0
170	R163	28.8	0.64676	0.19308	0.47616	0.98868	0	0	0	1	0
171	R163	33.6	0.64339	0.28413	0.52253	0.99211	0	0	0	1	0
172	R163	4.8	0.05407	0.00125	0.16349	0.98760	0	0	0	1	0
173	R163	14.4	0.42262	0.10167	0.63106	0.98415	0	0	0	1	0
174	R163	24.0	0.61713	0.10723	0.63825	0.99285	0	0	0	1	0
175	R163	19.2	0.57239	0.13252	0.61379	0.99196	0	0	0	1	0
176	R163	9.6	0.24398	0.10535	0.42143	0.99109	0	0	0	1	0
177	R163	12.0	0.38643	0.12133	0.52482	1.00000	0	0	0	1	0
178	R163	0.0	0.00301	0.01808	0.04492	0.98985	0	0	0	1	0
179	R163	38.4	0.63648	0.32274	0.60655	0.98840	0	0	0	1	0
180	R163	43.2	0.63092	0.52305	0.66663	0.98644	0	0	0	1	0
181	R163	0.0	0.00544	0.02870	0.03491	0.98760	0	0	0	1	0
182	R163	28.8	0.59710	0.43658	0.72592	0.96827	0	0	0	1	0
183											

	R163	7.2	0.27164	0.06144	0.56872	0.98986	0	0	0	1	0
184	R163	9.6	0.43579	0.14796	0.61109	0.99094	0	0	0	1	0
185	R163	4.8	0.10601	0.02011	0.33373	0.98088	0	0	0	1	0
186	R163	19.2	0.58243	0.06657	0.69106	0.97514	0	0	0	1	0
187	R163	33.6	0.55860	0.65785	0.52460	0.97439	0	0	0	1	0
188	R163	14.4	0.58905	0.13231	0.46794	0.97174	0	0	0	1	0
189	R163	12.0	0.53521	0.14117	0.45477	0.97895	0	0	0	1	0
190	R163	24.0	0.59524	0.28006	0.50184	0.97186	0	0	0	1	0
191	R163	0.0	0.00232	0.01877	0.07615	0.89774	0	0	0	1	0
192	R163	0.0	0.00200	0.02346	0.05512	0.91021	0	0	0	1	0
193	R163	4.8	0.50296	0.26347	0.40012	0.91278	0	0	0	1	0
194	R163	4.8	0.27430	0.09515	0.23832	0.86271	0	0	0	1	0
195	R163	7.2	0.58863	0.22256	0.57026	0.91479	0	0	0	1	0
196	R163	7.2	0.63378	0.34332	0.35795	0.84472	0	0	0	1	0
197	R163	9.6	0.73738	0.38217	0.34541	0.74732	0	0	0	1	0
198	R163	12.0	0.72278	0.17817	0.44251	0.88930	0	0	0	1	0
199	R163	19.2	0.71580	0.28894	0.30517	0.89099	0	0	0	1	0
200	R163	24.0	0.68835	0.75768	0.53075	0.91195	0	0	0	1	0
201	R163	28.0	0.84120	0.67624	0.87295	0.85726	0	0	0	1	0
202	R163	28.8	1.00000	1.00000	0.54737	0.86176	0	0	0	1	0
203	R163	33.6	0.82269	0.98914	0.81746	0.90049	0	0	0	1	0
204	R163	9.6	0.60119	0.14957	0.26468	0.85639	0	0	0	1	0
205	R163	28.0	0.78983	0.44988	0.89281	0.89827	0	0	0	1	0
206	R163	12.0	0.56508	0.14567	0.31945	0.85529	0	0	0	1	0
207	R163	33.6	0.87638	0.45896	1.00000	0.86531	0	0	0	1	0
208	R164	5.2	0.31049	0.03969	0.24748	1.00000	0	0	0	0	1
209	R164	0.0	0.04352	0.03390	0.01759	0.99876	0	0	0	0	1
210	R164	15.6	0.95174	0.55251	0.68089	0.92783	0	0	0	0	1
211	R164	10.4	0.74243	0.12933	0.65940	0.98129	0	0	0	0	1
212	R164	36.4	0.81674	0.96062	0.79095	0.83940	0	0	0	0	1
213	R164	7.8	0.61266	0.15679	0.64514	0.98124	0	0	0	0	1
214	R164	15.6	1.00000	0.52214	0.72970	0.95347	0	0	0	0	1
215	R164	13.0	0.98290	0.27435	0.71865	0.96288	0	0	0	0	1
216	R164	7.8	0.67640	0.18140	0.65725	0.97293	0	0	0	0	1
217	R164	5.2	0.57544	0.10404	0.42311	0.96770	0	0	0	0	1
218	R164	36.4	0.40771	0.80206	0.88134	0.82334	0	0	0	0	1
219	R164	20.8	0.59521	0.39875	0.74886	0.94630	0	0	0	0	1
220	R164	31.2	0.44027	0.94826	0.88958	0.80610	0	0	0	0	1
221	R164	31.2	0.66536	0.57477	0.83128	0.91446	0	0	0	0	1
222	R164	31.2	0.49419	1.00000	0.86608	0.76694	0	0	0	0	1
223	R164	20.8	0.77866	0.83898	0.79683	0.89449	0	0	0	0	1
224	R164	13.0	0.69480	0.23874	0.69401	0.97594	0	0	0	0	1
225	R164	0.0	0.06031	0.04178	0.02096	0.97481	0	0	0	0	1
226	R164	26.0	0.63199	0.97877	0.83295	0.85066	0	0	0	0	1
227	R164	0.0	0.08307	0.06022	0.00728	0.96954	0	0	0	0	1
228	R164	5.2	0.80409	0.13725	0.52592	0.96731	0	0	0	0	1
229											

	R164	15.6	0.77088	0.28586	0.63328	0.94557	0	0	0	0	1
230	R164	26.0	0.70318	0.56125	0.70399	0.90732	0	0	0	0	1
231	R164	13.0	0.87894	0.49834	0.69134	0.92288	0	0	0	0	1
232	R164	7.8	0.73394	0.13427	0.54365	0.95921	0	0	0	0	1
233	R164	20.8	0.91997	0.87430	0.76802	0.90005	0	0	0	0	1
234	R164	10.4	0.72557	0.22917	0.63413	0.96212	0	0	0	0	1
235	R164	10.4	0.59040	0.17875	0.61846	0.95848	0	0	0	0	1
236	R164	46.8	0.78163	0.91669	0.93512	0.84957	0	0	0	0	1
237	R164	41.6	0.49938	0.75262	0.91548	0.80806	0	0	0	0	1
238	R164	18.0	0.74636	0.51392	0.70719	0.88638	0	0	0	0	1
239	R164	28.8	0.51907	0.92010	0.91664	0.87018	0	0	0	0	1
240	R164	36.0	0.39906	0.82725	0.92563	0.71789	0	0	0	0	1
241	R164	10.8	0.42097	0.10778	0.76111	0.97517	0	0	0	0	1
242	R164	21.6	0.64284	0.54343	0.76170	0.88181	0	0	0	0	1
243	R164	0.0	0.05091	0.03763	0.00325	0.96543	0	0	0	0	1
244	R164	7.2	0.67202	0.12992	0.63126	0.96398	0	0	0	0	1
245	R164	14.4	0.61536	0.26787	0.71837	0.94238	0	0	0	0	1
246	R164	43.2	0.38937	0.75655	0.98958	0.69575	0	0	0	0	1
247	R164	14.4	0.62639	0.21291	0.77263	0.95563	0	0	0	0	1
248	R164	43.2	0.36432	0.78846	1.00000	0.71706	0	0	0	0	1
249	R164	7.2	0.40531	0.12866	0.61356	0.97066	0	0	0	0	1
250	R164	21.6	0.78461	0.42455	0.73778	0.87833	0	0	0	0	1
251	R164	18.0	0.64931	0.37749	0.76603	0.94590	0	0	0	0	1
252	R164	0.0	0.03983	0.03773	0.01856	0.96882	0	0	0	0	1
253	R164	28.8	0.56028	0.74778	0.92749	0.88176	0	0	0	0	1
254	R164	36.0	0.41961	0.86599	0.95705	0.82288	0	0	0	0	1
255	R164	10.8	0.37718	0.14004	0.74562	0.96211	0	0	0	0	1
256	R164	9.0	0.45894	0.15177	0.78709	0.95851	0	0	0	0	1
257	R164	0.0	0.04323	0.05055	0.01356	0.96185	0	0	0	0	1
258	R164	21.6	0.40920	0.30492	0.73622	0.91308	0	0	0	0	1
259	R164	5.4	0.11512	0.10649	0.24005	0.97260	0	0	0	0	1
260	R164	25.2	0.35214	0.27143	0.82548	0.90979	0	0	0	0	1
261	R164	3.6	0.06205	0.07168	0.27202	0.97822	0	0	0	0	1
262	R164	10.8	0.34721	0.13698	0.76800	0.98087	0	0	0	0	1
263	R164	14.4	0.37465	0.19437	0.70134	0.95596	0	0	0	0	1
264	R164	18.0	0.28535	0.26761	0.70885	0.95561	0	0	0	0	1
265	R164	7.2	0.03495	0.08396	0.60175	0.98394	0	0	0	0	1
266	R164	28.8	0.19442	0.49383	0.88587	0.90105	0	0	0	0	1
267	R164	36.0	0.21697	0.55132	0.87796	0.90250	0	0	0	0	1
268	R165	5.4	0.01851	0.03939	0.04020	1.00000	0	0	0	0	0
269	R165	0.0	0.02224	0.04620	0.06365	0.99353	0	0	0	0	0
270	R165	0.0	0.01443	0.03414	0.03932	0.98824	0	0	0	0	0
271	R165	25.2	0.66393	0.72507	0.90896	0.76164	0	0	0	0	0
272	R165	18.0	0.64101	0.62474	0.83339	0.83021	0	0	0	0	0
273	R165	3.6	0.00792	0.02877	0.04637	0.96548	0	0	0	0	0
274	R165	21.6	0.61748	0.66920	0.93620	0.75134	0	0	0	0	0
275											

	R165	7.2	0.20556	0.12094	0.12560	0.96696	0	0	0	0	0
276	R165	9.0	0.16973	0.11256	0.12214	0.96516	0	0	0	0	0
277	R165	18.0	0.59526	0.47131	0.78807	0.92216	0	0	0	0	0
278	R165	10.8	0.49714	0.23985	0.61426	0.95302	0	0	0	0	0
279	R165	9.0	0.50343	0.22140	0.58483	0.95525	0	0	0	0	0
280	R165	7.2	0.38533	0.18000	0.18017	0.95450	0	0	0	0	0
281	R165	25.2	0.67540	0.48676	0.76985	0.91313	0	0	0	0	0
282	R165	14.4	0.45812	0.23922	0.63124	0.95854	0	0	0	0	0
283	R165	5.4	0.05509	0.03883	0.05609	0.95563	0	0	0	0	0
284	R165	9.0	0.39409	0.15633	0.25642	0.95309	0	0	0	0	0
285	R165	0.0	0.01835	0.02454	0.04987	0.95929	0	0	0	0	0
286	R165	21.6	0.74046	0.43860	0.77538	0.93325	0	0	0	0	0
287	R165	5.4	0.12482	0.08132	0.08319	0.96299	0	0	0	0	0
288	R165	10.8	0.57290	0.24672	0.63075	0.95560	0	0	0	0	0
289	R165	10.8	0.23868	0.13364	0.28997	0.96632	0	0	0	0	0
290	R165	14.4	0.51166	0.43429	0.84121	0.94225	0	0	0	0	0
291	R165	14.4	0.50065	0.39817	0.64515	0.94759	0	0	0	0	0
292	R165	3.6	0.03201	0.02938	0.06476	0.97730	0	0	0	0	0
293	R165	7.2	0.04277	0.02979	0.05840	0.97524	0	0	0	0	0
294	R165	21.6	0.53705	0.55261	0.85548	0.83016	0	0	0	0	0
295	R165	18.0	0.54526	0.29594	0.67577	0.96833	0	0	0	0	0
296	R165	3.6	0.01158	0.03646	0.04861	0.97269	0	0	0	0	0
297	R165	25.2	0.62062	0.72071	1.00000	0.75919	0	0	0	0	0
298	R165	0.0	0.01779	0.02861	0.01851	0.92828	0	0	0	0	0
299	R165	6.4	0.48225	0.27560	0.06238	0.92533	0	0	0	0	0
300	R165	3.2	0.06012	0.05945	0.02525	0.92215	0	0	0	0	0
301	R165	3.2	0.04756	0.05561	0.04366	0.92932	0	0	0	0	0
302	R165	19.2	1.00000	0.97735	0.60335	0.78745	0	0	0	0	0
303	R165	16.0	0.82381	0.70423	0.61483	0.94045	0	0	0	0	0
304	R165	8.0	0.27454	0.20951	0.05461	0.95892	0	0	0	0	0
305	R165	22.4	0.98804	1.00000	0.60243	0.86328	0	0	0	0	0
306	R165	12.8	0.59549	0.52705	0.36331	0.95378	0	0	0	0	0
307	R165	0.0	0.01083	0.01254	0.02409	0.95015	0	0	0	0	0
308	R165	4.8	0.17096	0.11468	0.00450	0.93698	0	0	0	0	0
309	R165	22.4	0.97534	0.94024	0.76724	0.74048	0	0	0	0	0
310	R165	6.4	0.17059	0.15788	0.05390	0.96334	0	0	0	0	0
311	R165	6.4	0.41393	0.24362	0.08235	0.96000	0	0	0	0	0
312	R165	9.6	0.65273	0.41473	0.33241	0.95013	0	0	0	0	0
313	R165	9.6	0.74021	0.44297	0.51571	0.93475	0	0	0	0	0
314	R165	19.2	0.91980	0.83844	0.70394	0.82899	0	0	0	0	0
315	R165	22.4	0.88650	0.83884	0.68957	0.79813	0	0	0	0	0
316	R165	9.6	0.34500	0.24596	0.11214	0.97163	0	0	0	0	0
317	R165	16.0	0.89906	0.73447	0.67128	0.88844	0	0	0	0	0
318	R165	16.0	0.99025	0.72750	0.64431	0.87127	0	0	0	0	0
319	R165	4.8	0.19735	0.13103	0.02943	0.96261	0	0	0	0	0
320	R165	0.0	0.01205	0.03620	0.03319	0.95523	0	0	0	0	0
321											

	R165	12.8	0.79224	0.54543	0.63159	0.90068	0	0	0	0	0
322	R165	12.8	0.73558	0.53968	0.63810	0.92755	0	0	0	0	0
323	R165	8.0	0.50374	0.29988	0.22017	0.94998	0	0	0	0	0
324	R165	8.0	0.40899	0.27034	0.09287	0.94619	0	0	0	0	0
325	R165	3.2	0.01286	0.03160	0.07768	0.94225	0	0	0	0	0
326	R165	4.8	0.02167	0.03670	0.02802	0.95268	0	0	0	0	0
327	R165	19.2	0.86617	0.48594	0.65368	0.92310	0	0	0	0	0
328	R165	0.0	0.02367	0.02426	0.03407	0.97921	0	0	0	0	0
329	R165	16.0	0.08714	0.04632	0.00397	0.98901	0	0	0	0	0
330	R165	19.2	0.43925	0.27103	0.47545	0.97414	0	0	0	0	0
331	R165	19.2	0.47082	0.36409	0.72798	0.96554	0	0	0	0	0
332	R165	4.8	0.01326	0.03730	0.03593	0.98784	0	0	0	0	0
333	R165	0.0	0.01402	0.01786	0.04777	0.99662	0	0	0	0	0
334	R165	12.8	0.27082	0.13617	0.10365	0.99050	0	0	0	0	0

The SAS System

The REG Procedure
Model: MODEL1
Dependent Variable: HR_norm

Number of Observations Read	334
Number of Observations Used	334

Analysis of Variance					
Source	DF	Sum of Squares	Mean Square	F Value	Pr > F
Model	6	1.16269	0.19378	49.22	<.0001
Error	327	1.28743	0.00394		
Corrected Total	333	2.45012			

Root MSE	0.06275	R-Square	0.4745
Dependent Mean	0.90845	Adj R-Sq	0.4649
Coeff Var	6.90694		

Parameter Estimates					
Variable	DF	Parameter Estimate	Standard Error	t Value	Pr > |t|
Intercept	1	0.96171	0.00803	119.71	<.0001
Q	1	-0.00297	0.00022221	-13.35	<.0001
R153dummy	1	-0.00502	0.01138	-0.44	0.6594
R154dummy	1	0.04803	0.01266	3.79	0.0002
R162dummy	1	-0.06712	0.01223	-5.49	<.0001
R163dummy	1	0.03394	0.01186	2.86	0.0045
R164dummy	1	0.00870	0.01126	0.77	0.4403

The SAS System

The REG Procedure
Model: MODEL1
Dependent Variable: HR_norm

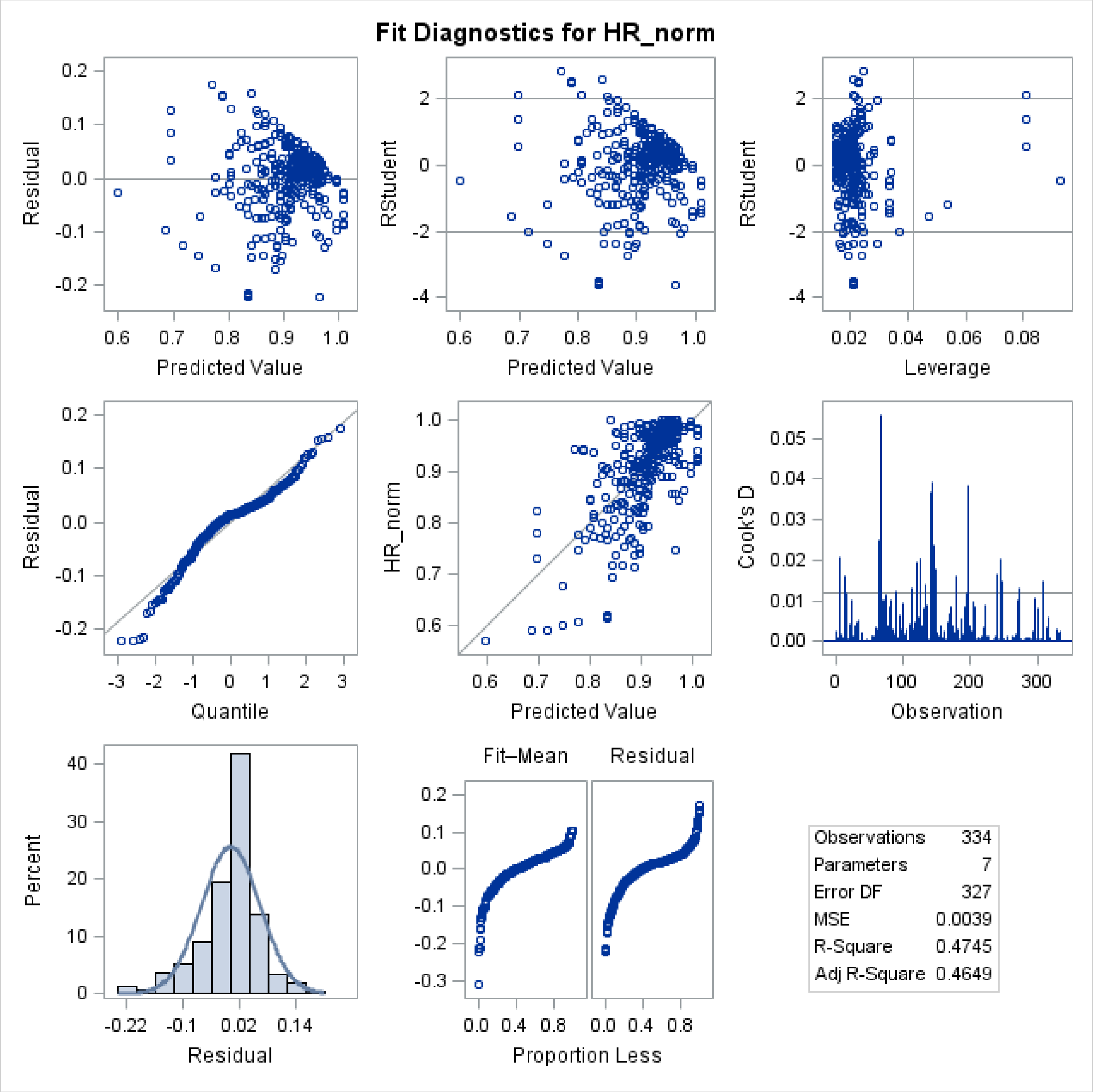

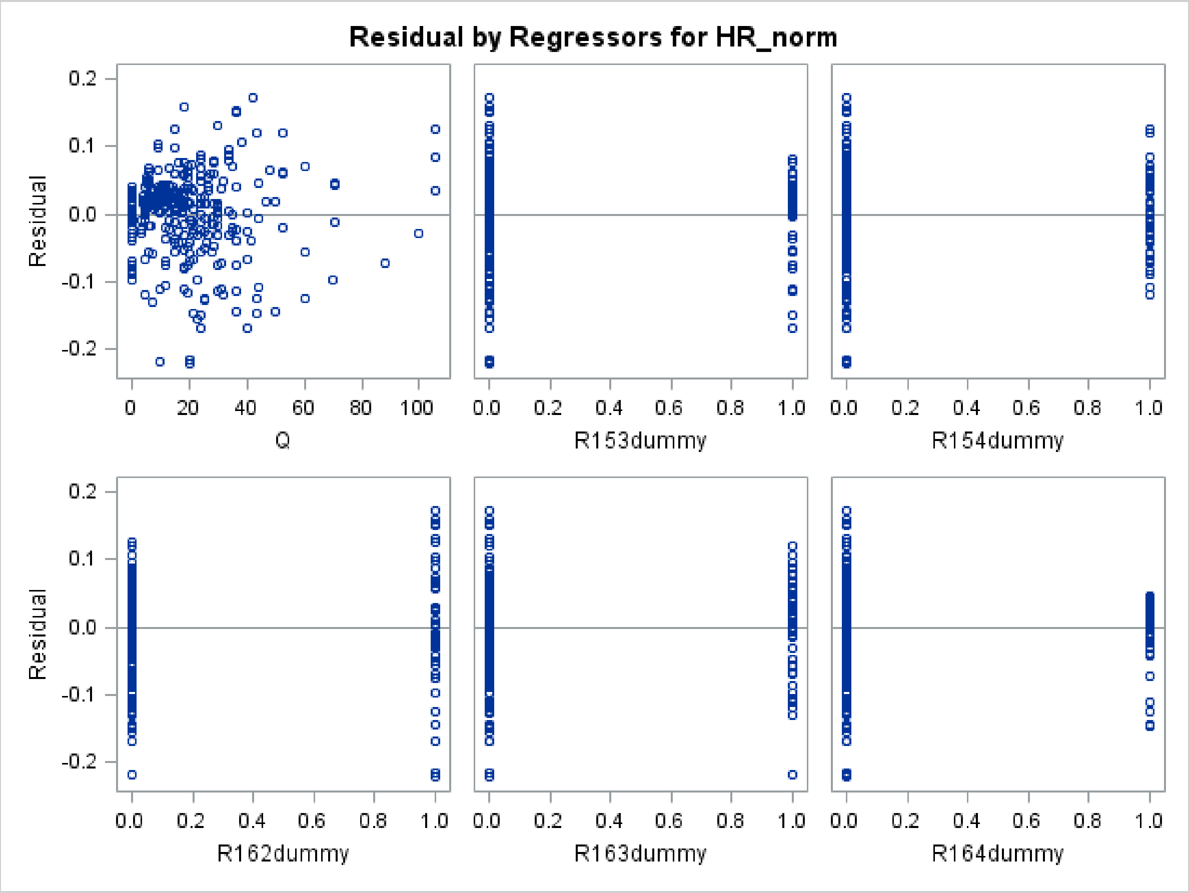
Residual by Regressors for HR_norm
Residual
0.2
0.1
0.0
-0.1
-0.2
0
20
40
60
80
100
Q
0.0
0.2
0.4
0.6
0.8
1.0
R153dummy
R154dummy
R162dummy
R163dummy
R164dummy

The SAS System

The REG Procedure
Model: MODEL1
Dependent Variable: HR_norm

Number of Observations Read	334
Number of Observations Used	334

Analysis of Variance					
Source	DF	Sum of Squares	Mean Square	F Value	Pr > F
Model	6	0.61351	0.10225	18.21	<.0001
Error	327	1.83661	0.00562		
Corrected Total	333	2.45012			

Root MSE	0.07494	R-Square	0.2504
Dependent Mean	0.90845	Adj R-Sq	0.2366
Coeff Var	8.24958		

Parameter Estimates					
Variable	DF	Parameter Estimate	Standard Error	t Value	Pr > |t|
Intercept	1	0.96288	0.01116	86.26	<.0001
A	1	-0.08328	0.01599	-5.21	<.0001
R153dummy	1	-0.02289	0.01366	-1.68	0.0948
R154dummy	1	-0.02814	0.01374	-2.05	0.0413
R162dummy	1	-0.12147	0.01445	-8.40	<.0001
R163dummy	1	0.02530	0.01417	1.79	0.0751
R164dummy	1	-0.00273	0.01344	-0.20	0.8392

The SAS System

The REG Procedure
Model: MODEL1
Dependent Variable: HR_norm

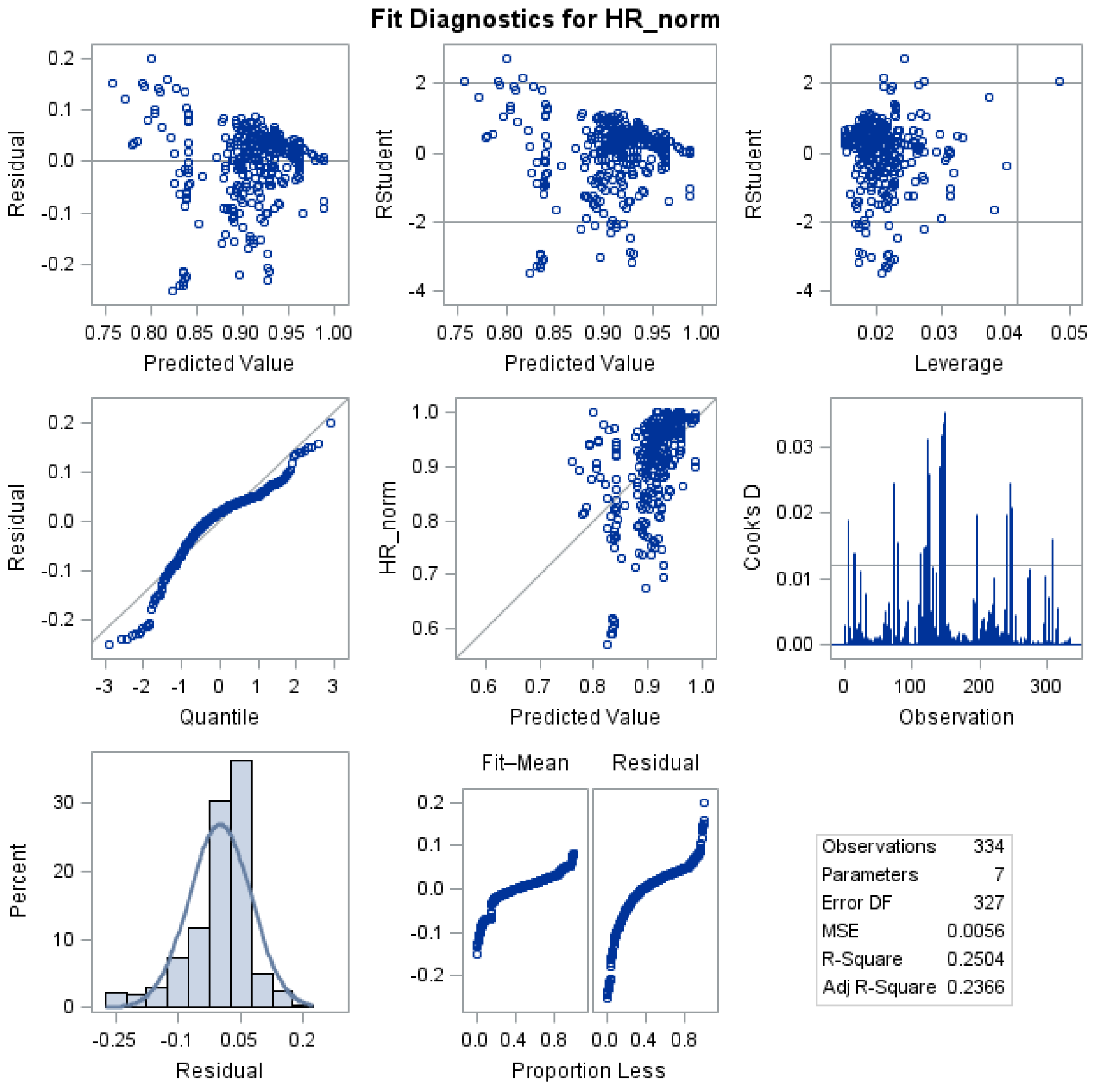

Observations	334
Parameters	7
Error DF	327
MSE	0.0056
R-Square	0.2504
Adj R-Square	0.2366

Residual by Regressors for HR_norm

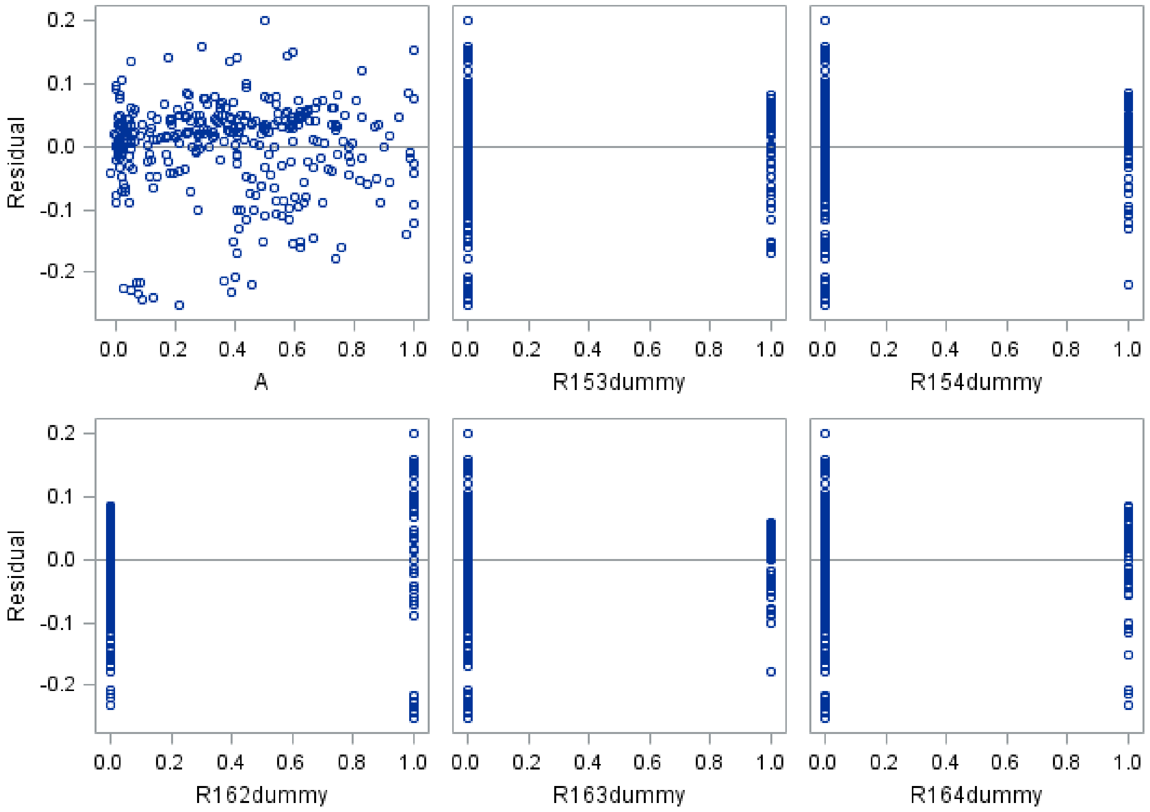

The SAS System

The REG Procedure
Model: MODEL1
Dependent Variable: HR_norm

Number of Observations Read	334
Number of Observations Used	334

Analysis of Variance					
Source	DF	Sum of Squares	Mean Square	F Value	Pr > F
Model	6	1.43366	0.23894	76.87	<.0001
Error	327	1.01646	0.00311		
Corrected Total	333	2.45012			

Root MSE	0.05575	R-Square	0.5851
Dependent Mean	0.90845	Adj R-Sq	0.5775
Coeff Var	6.13718		

Parameter Estimates					
Variable	DF	Parameter Estimate	Standard Error	t Value	Pr > |t|
Intercept	1	0.99033	0.00763	129.84	<.0001
B	1	-0.19671	0.01112	-17.69	<.0001
R153dummy	1	-0.02851	0.01012	-2.82	0.0052
R154dummy	1	-0.00841	0.01023	-0.82	0.4118
R162dummy	1	-0.07761	0.01067	-7.27	<.0001
R163dummy	1	0.00724	0.01050	0.69	0.4905
R164dummy	1	0.00583	0.00996	0.59	0.5589

The SAS System

The REG Procedure
Model: MODEL1
Dependent Variable: HR_norm

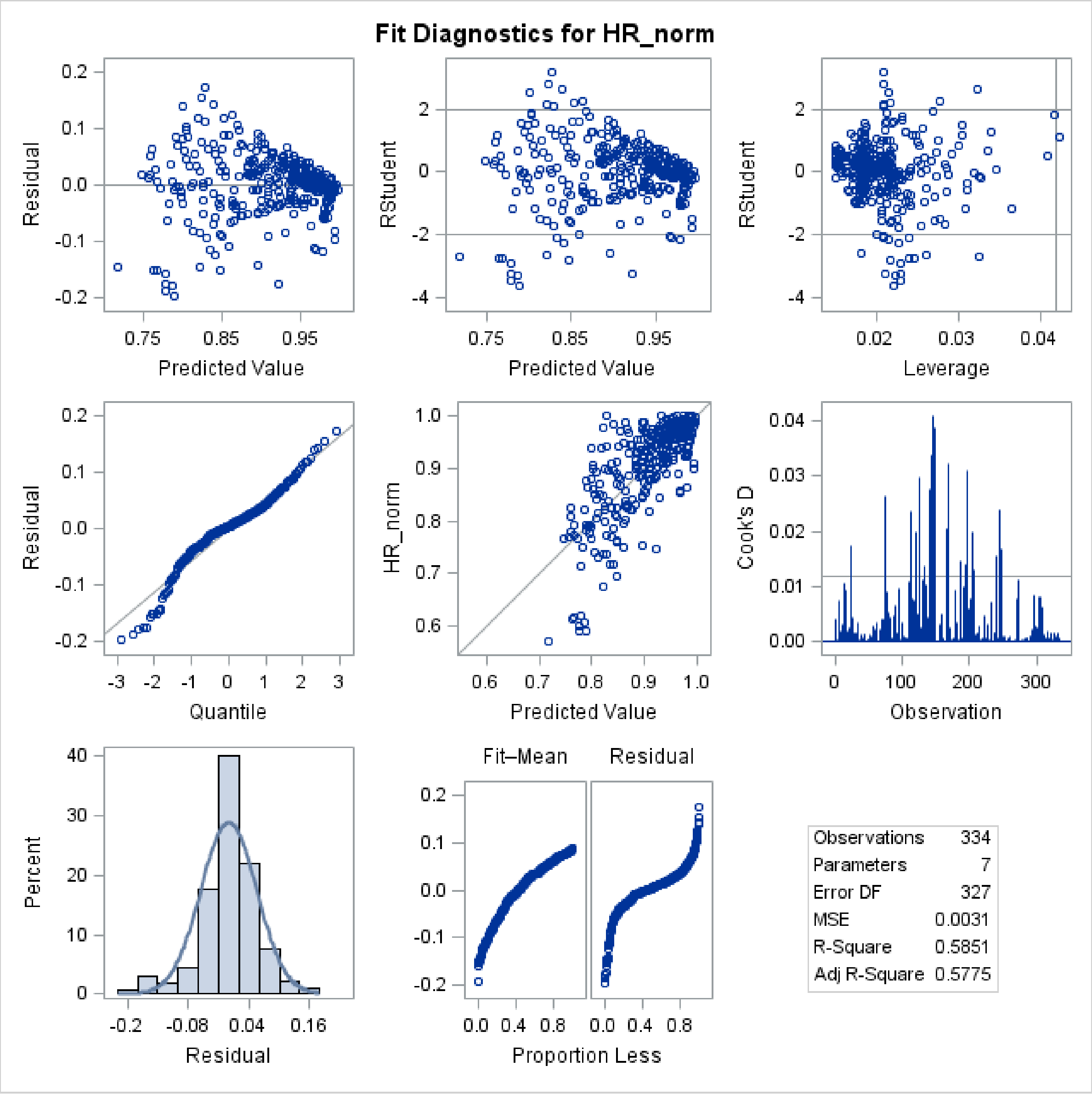

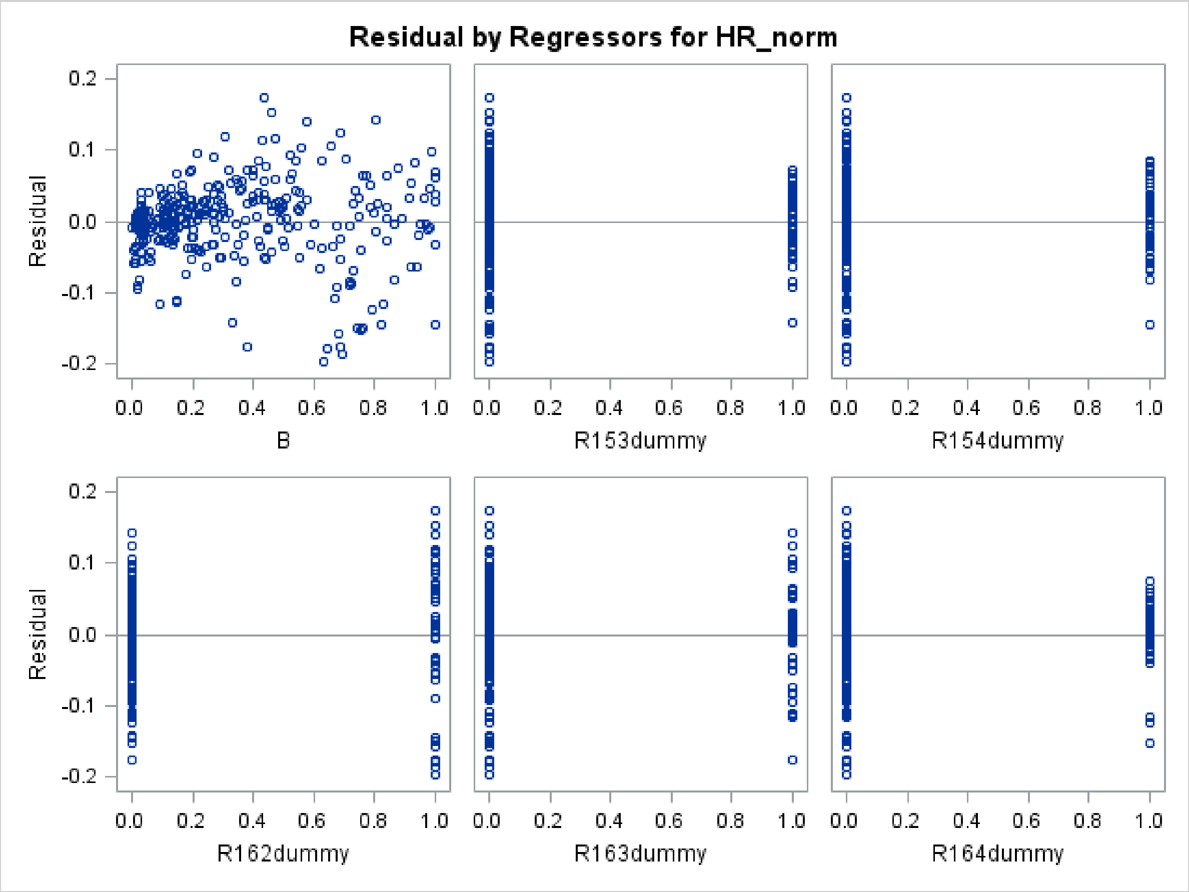
Residual by Regressors for HR_norm
Residual
0.2
0.1
0.0
-0.1
-0.2
0.0 0.2 0.4 0.6 0.8 1.0
B
R153dummy
R154dummy
R162dummy
R163dummy
R164dummy

The SAS System

The REG Procedure
Model: MODEL1
Dependent Variable: HR_norm

Number of Observations Read	334
Number of Observations Used	334

Analysis of Variance					
Source	DF	Sum of Squares	Mean Square	F Value	Pr > F
Model	6	0.66184	0.11031	20.17	<.0001
Error	327	1.78828	0.00547		
Corrected Total	333	2.45012			

Root MSE	0.07395	R-Square	0.2701
Dependent Mean	0.90845	Adj R-Sq	0.2567
Coeff Var	8.14031		

Parameter Estimates					
Variable	DF	Parameter Estimate	Standard Error	t Value	Pr > |t|
Intercept	1	0.96137	0.01044	92.04	<.0001
C	1	-0.08925	0.01473	-6.06	<.0001
R153dummy	1	-0.01955	0.01341	-1.46	0.1460
R154dummy	1	0.00566	0.01435	0.39	0.6937
R162dummy	1	-0.07741	0.01479	-5.23	<.0001
R163dummy	1	0.02766	0.01401	1.98	0.0491
R164dummy	1	0.01510	0.01388	1.09	0.2774

The SAS System

The REG Procedure
Model: MODEL1
Dependent Variable: HR_norm

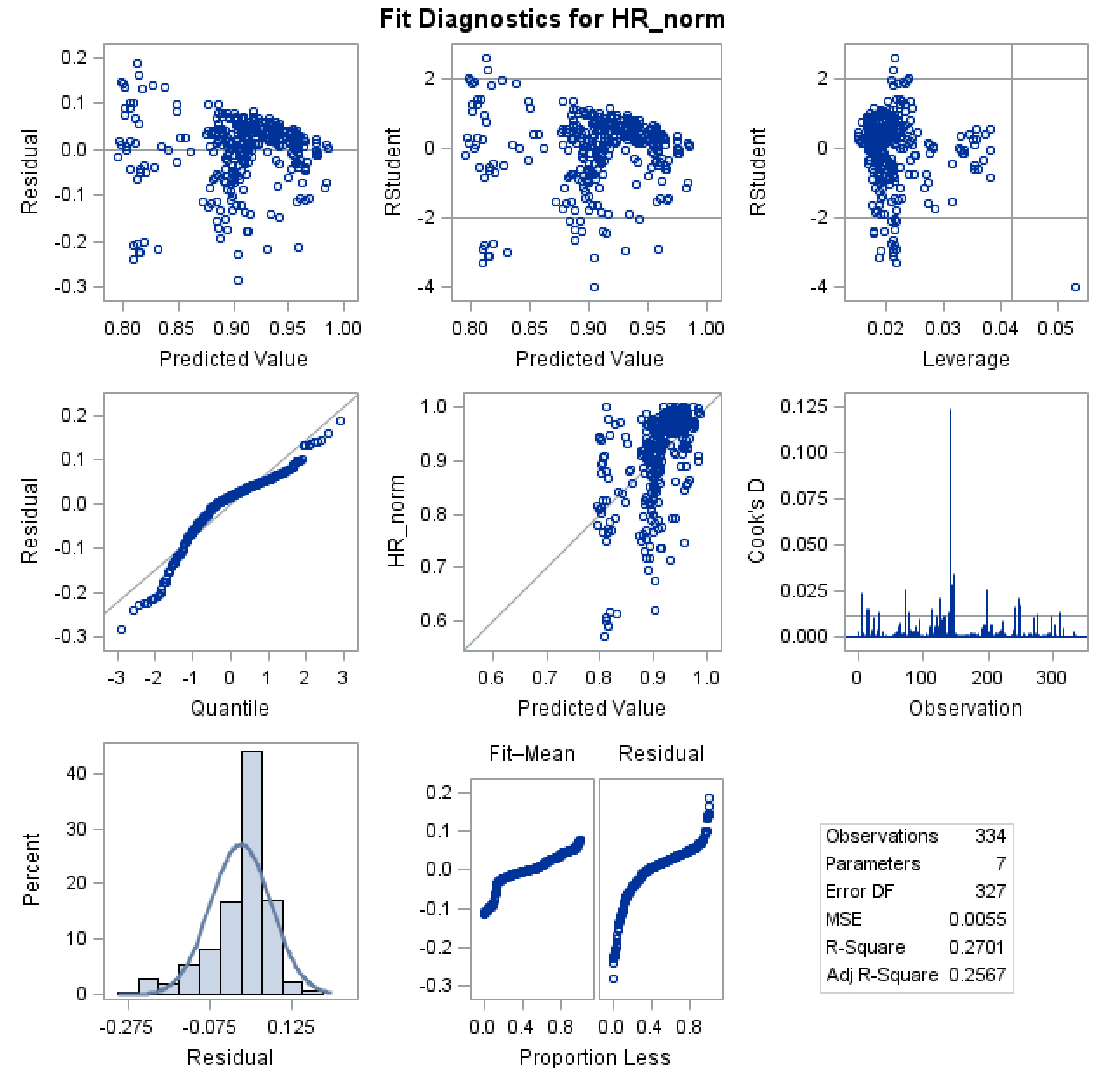

Observations	334
Parameters	7
Error DF	327
MSE	0.0055
R-Square	0.2701
Adj R-Square	0.2567

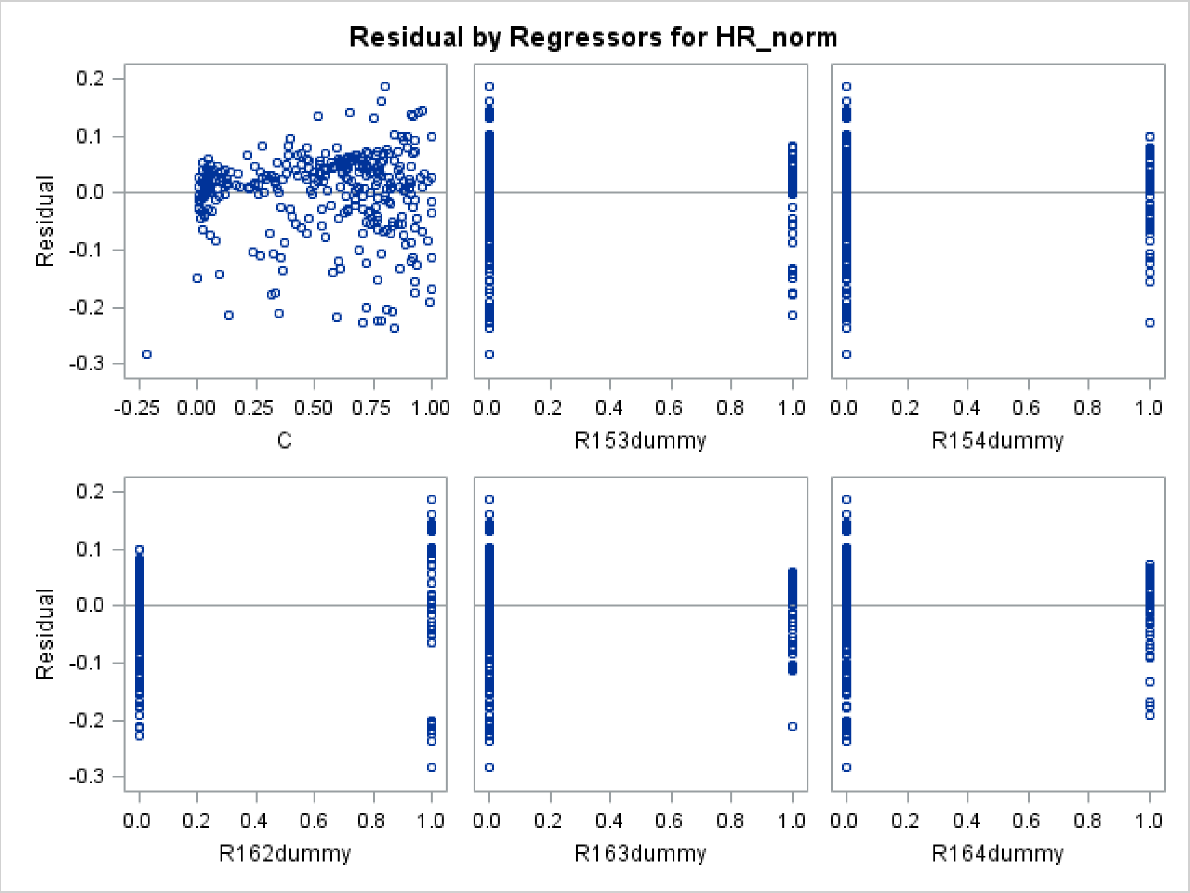
Residual by Regressors for HR_norm
Residual
0.2
0.1
0.0
-0.1
-0.2
-0.3
-0.25
0.00
0.25
0.50
0.75
1.00
C
0.0
0.2
0.4
0.6
0.8
1.0
R153dummy
R154dummy
R162dummy
R163dummy
R164dummy
